# Circulating succinate-modifying metabolites accurately classify and reflect the status of fumarate hydratase–deficient renal cell carcinoma

**DOI:** 10.1172/JCI165028

**Published:** 2023-06-01

**Authors:** Liang Zheng, Zi-Ran Zhu, Tal Sneh, Wei-Tuo Zhang, Zao-Yu Wang, Guang-Yu Wu, Wei He, Hong-Gang Qi, Hang Wang, Xiao-Yu Wu, Jonatan Fernández-García, Ifat Abramovich, Yun-Ze Xu, Jin Zhang, Eyal Gottlieb

**Affiliations:** 1 Key Laboratory of Pediatric Hematology and Oncology, Ministry of Health, Pediatric Translational Medicine Institute, Shanghai Children’s Medical Center, School of Medicine, and; 2 Department of Urology, Renji Hospital, School of Medicine, Shanghai Jiaotong University, Shanghai, China.; 3 Fujian Branch of Shanghai Children’s Medical Center, Shanghai Jiaotong University School of Medicine, Fujian Children’s Hospital, Fuzhou, Fujian, China.; 4 Department of Pharmacology and Chemical Biology, Shanghai Jiao Tong University School of Medicine, Shanghai, China.; 5 Ruth and Bruce Rappaport Faculty of Medicine, Technion – Israel Institute of Technology, Haifa, Israel.; 6 Hongqiao International Institute of Medicine, Shanghai Tong Ren Hospital and Clinical Research Institute, Shanghai Jiao Tong University, Shanghai, China.; 7 Department of Pathology, and; 8 Department of Radiology, Renji Hospital, School of Medicine, Shanghai Jiaotong University, Shanghai, China.; 9 Department of Urology, the First Affiliated Hospital, School of Medicine, Zhejiang University, Hangzhou, China.; 10 Department of Urology, Zhongshan Hospital, Fudan University, Shanghai, China.; 11 Department of Cancer Biology, University of Texas MD Anderson Cancer Center, Houston, Texas, USA.

**Keywords:** Metabolism, Oncology, Cancer, Genetic diseases, Molecular diagnosis

## Abstract

Germline or somatic loss-of-function mutations of fumarate hydratase (FH) predispose patients to an aggressive form of renal cell carcinoma (RCC). Since other than tumor resection there is no effective therapy for metastatic FH-deficient RCC, an accurate method for early diagnosis is needed. Although MRI or CT scans are offered, they cannot differentiate FH-deficient tumors from other RCCs. Therefore, finding noninvasive plasma biomarkers suitable for rapid diagnosis, screening, and surveillance would improve clinical outcomes. Taking advantage of the robust metabolic rewiring that occurs in FH-deficient cells, we performed plasma metabolomics analysis and identified 2 tumor-derived metabolites, succinyl-adenosine and succinic-cysteine, as excellent plasma biomarkers for early diagnosis. These 2 molecules reliably reflected the FH mutation status and tumor mass. We further identified the enzymatic cooperativity by which these biomarkers are produced within the tumor microenvironment. Longitudinal monitoring of patients demonstrated that these circulating biomarkers can be used for reporting on treatment efficacy and identifying recurrent or metastatic tumors.

## Graphical abstract

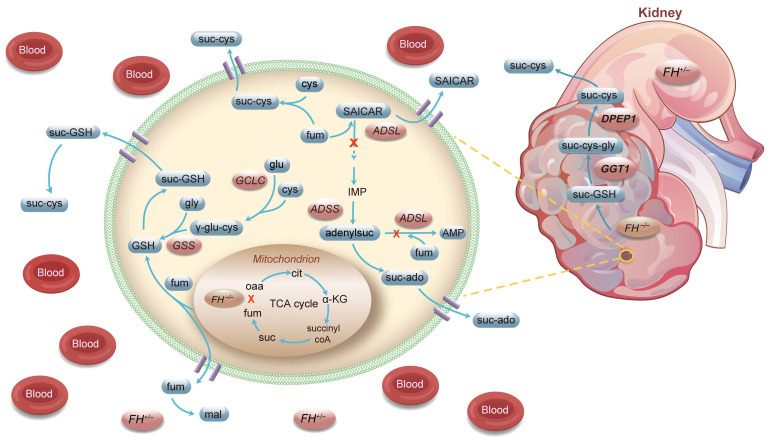

## Introduction

Renal cell carcinoma (RCC) is a remarkably heterogeneous malignancy that originates from renal tubular epithelial cells. It accounted for 2%–3% of cancers and caused more than 200,000 deaths worldwide in 2020 (1). Multiple subtypes of RCC have been characterized by distinct pathophysiologies, genetic alterations, clinical courses, and therapeutic responses (2). Fumarate hydratase–deficient (FH-deficient) RCC is a recently described unique subtype of RCC characterized by germline or somatic ablation of *FH*, with a coinciding somatic hit that affects the second *FH* allele in the tumor. Apart from loss of FH activity, FH-deficient RCC often features the CpG island methylator phenotype (CIMP) and CpG island methylation alongside the CDKN2A promoter (3, 4). FH-deficient RCC is a disease associated with hereditary leiomyomatosis and renal cell carcinoma (HLRCC), which manifests as cutaneous or uterine leiomyomas with or without aggressive RCC (5, 6). Given its high propensity to invade and metastasize and high rate of mortality, FH-deficient RCC is the most aggressive form of RCC. In the reported FH-deficient RCC cohorts, 65%–85% of patients were already at a metastatic stage at their initial diagnosis. More than 70% of the patients died of late-stage RCC within 5 years of diagnosis (7–10). To our knowledge, there is no effective screening or diagnosis method for the early detection of FH-deficient RCC. The most beneficial treatment is aggressive surgical resection. When tumors are relatively small and localized, unique surgical approaches for FH-deficient RCC can be taken. These include immediate resection upon observation, partial nephrectomy with wider margins or radical nephrectomy, regional lymph node dissection, and/or open surgery (11–15). Although less specific, systemic therapies targeting VEGF, EGFR, or mTOR along with immune checkpoint inhibitors have shown promising efficacy for metastatic FH-deficient tumors, and several clinical trials are ongoing (16–20). Hence, discovering early diagnostic biomarkers for FH-deficient RCC that would increase the prospects of detecting the disease in its localized stage would improve the clinical outcome of patients.

FH is a TCA cycle enzyme that converts fumarate to malate. It has been well established that FH loss impairs TCA cycle activity and thus causes fumarate accumulation, which induces various oncometabolic effects, including HIF activation caused by HIF prolyl hydroxylase inhibition (21–24), hypermethylation caused by inhibition of histone and DNA demethylases (25), an antioxidant response mediated by NRF2 activation (26), reversal of the urea cycle (27), and induction of the DNA damage response (DDR) caused by homologous recombination inhibition (28). All these tumorigenic effects are a consequence of the direct reactivity of fumarate in both enzymatic and nonenzymatic reactions. Therefore, FH-deficient RCC is genuinely a metabolic disease in which aberrant metabolic rewiring elicits malignant transformation and facilitates the phenotypic evolution of tumors (29).

Identifying FH-deficient RCC is a challenging task. It requires multiple procedures, such as radiological imaging, histopathological evaluation, IHC staining, and genetic counseling. The classic morphological hallmark is prominent CMV inclusion–type eosinophilic nucleoli surrounded by clear nuclei halos (10). However, these features are also characteristics of medullary carcinoma and collecting duct carcinoma, hindering the discriminatory accuracy of a morphology-based diagnosis. Fumarate-mediated posttranslational modification of proteins via cystine succination was introduced as a potential biomarker for FH-deficient tumors (30). IHC performed on tumor biopsies with antibodies against S-(2-succino)-cysteine (2SC) in combination with anti-FH antibody is a sensitive assay, although its specificity still needs to be validated (30–32). To date, genetic sequencing remains the benchmark for characterizing FH-deficient tumors. Regardless of specificity and accuracy, both IHC and genetic analyses require invasive procedures to obtain biopsy materials. Furthermore, while IHC and genetic analyses are useful for characterizing FH-deficient tumors, they are not suitable for screening and early diagnosis, for determining the efficacy of treatment, for detecting minimal residual disease (MRD), or for monitoring to detect recurrent disease. Considering the need for early tumor detection and early clinical intervention, it is not surprising that under the National Cancer Institute’s (NCI’s) Physician Data Query (PDQ) for FH-deficient RCC, it is stated that “the development of diagnostic blood-based tests or imaging tools that permit cost-effective surveillance of the kidneys of patients with HLRCC would have a major positive effect on the outcomes of these individuals” (33).

This study is the first comprehensive clinical research of noninvasive biomarkers in the plasma metabolome in a large cohort of patients with FH-deficient RCC (*n* = 77). Taking advantage of highly sensitive MS-based metabolic profiling, we identified 2 robust circulating biomarkers, succinic-cysteine (suc-cys) and succinyl-adenosine (suc-ado), which can sensitively and specifically reflect FH status and tumor burden in RCC. We separately validated the superior screening and diagnostic power of these markers with receiver operating characteristic (ROC) area under curve (AUC) (ROCAUC) analysis; the markers achieved a ROCAUC of 0.98 for distinguishing FH-mutant (FH-MT) RCC from nontumor normal control (NC) or FH–wild type (FH-WT) RCC samples. We further identified the mechanisms underlying the excellent performance of suc-cys and suc-ado, but not other circulating metabolites, as liquid biopsy biomarkers. Finally, via longitudinal assessment of the dynamics of these plasma biomarkers, we demonstrated their powerful capacity to monitor treatment efficacy in real time and identify recurrent or metastatic tumors.

## Results

### Clinicopathological characteristics of the renal cell carcinoma cohort.

The SHJTUCMRJ study cohort consisted of 252 participants from 16 centers: patients with FH-deficient (FH-MT) RCC spanning the full spectrum of stages (I, II, III and IV; *n* = 77) were from our established cohort (34); 70 of 77 of the patients with FH-MT carried germline mutations, whereas 7 were sporadic cases involving missense, nonsense, frameshift, and large-scale deletion. Of all patients with FH-MT, 47 had a documented family history of RCC (Supplemental Table 1; supplemental material available online with this article; https://doi.org/10.1172/JCI165028DS1). Patients with FH-WT RCC (*n* = 88) were newly recruited and included individuals with various subtypes of RCC: papillary RCC (pRCC) type 2 (*n* = 33), clear cell RCC (ccRCC) (*n* = 26), pRCC type 1 (*n* = 21), *Xp11.2*-translocation RCC (*n* = 2), and uncharacterized pRCC (*n* = 6); NC (no tumor) individuals were newly recruited and were age and sex matched (*n* = 87). A total of *n* = 268 plasma samples were used in the study. Samples (*n* = 30) from 10 patients with FH-MT RCC, 10 patients with FH-WT RCC, and 10 NC individuals were selected as the discovery set. The separate validation set (*n* = 238) comprised 67 FH-MT samples, 78 FH-WT samples, and 77 NC samples (Supplemental Figure 1, Supplemental Figure 2A, and Supplemental Table 1).

**Figure 1:**
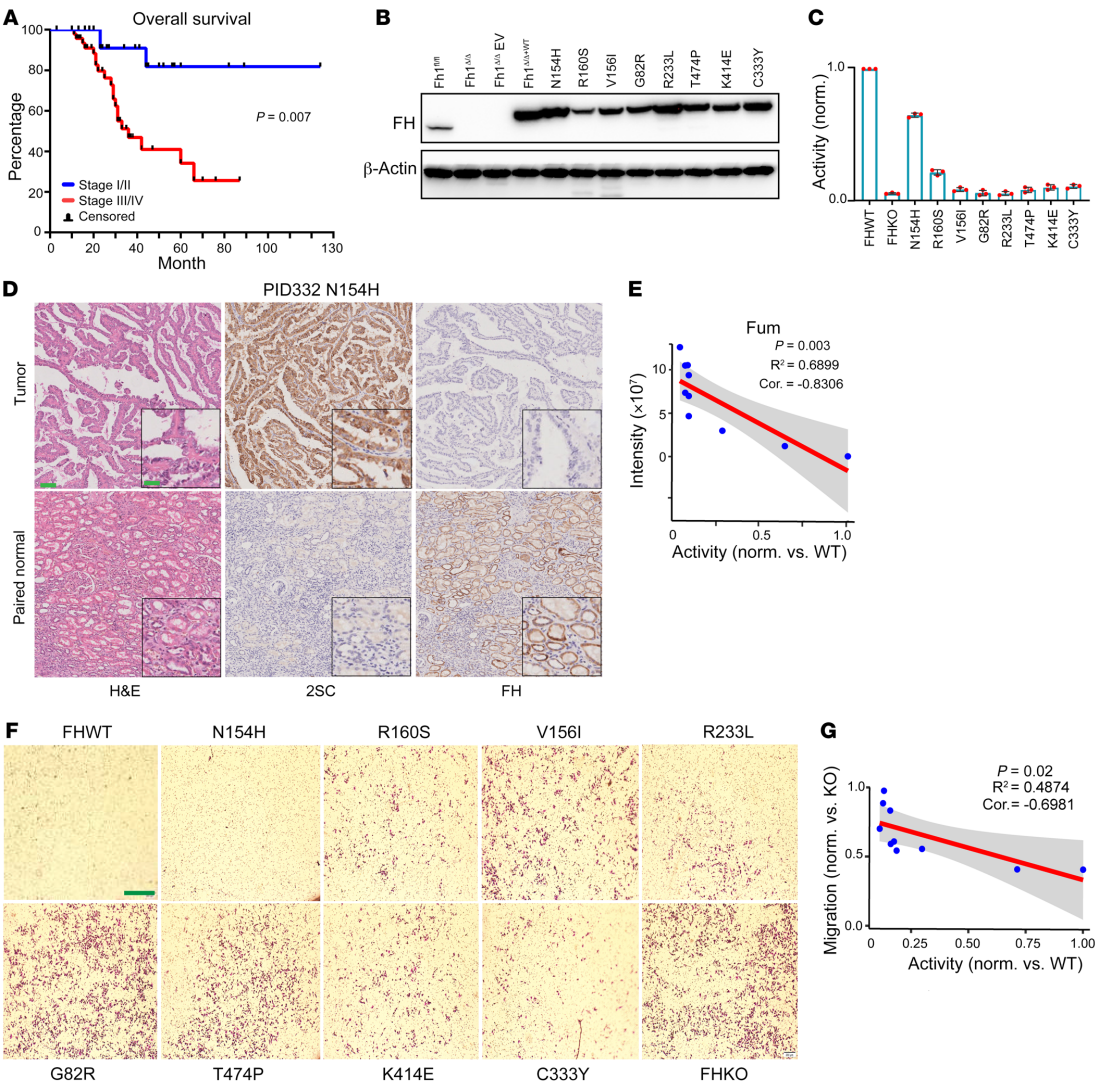
Pathological characteristics of the RCC cohort. (**A**) Kaplan-Meier analysis of OS following diagnosis for patients with stage I/II FH-deficient RCC versus those with stage III/IV FH-deficient RCC (log-rank test, *P* = 0.007). (**B**) Expression of mutant FH proteins in FH-null mouse cells. (**C**) Relative in vitro enzymatic activity of mutant FH proteins normalized to WT activity. The experiments were performed independently 3 times. All data are presented as the mean ± SEM. (**D**) H&E and IHC staining of 2SC and FH tissue from patient PID332 p.N154H. Scale bars: 200 μm and 50 μm (insets). (**E**) Negative correlation (cor.) between intracellular fumarate (Fum) levels and variant FH enzymatic activity. (**F**) Migratory capability of cells in Matrigel. Scale bar: 600 μm. (**G**) Negative correlation between cellular migration and variant FH enzymatic activity in cells. norm., normalized.

We first performed Kaplan-Meier survival analysis of 77 patients with FH-MT RCC, which showed that the average survival of patients with stage I/II disease was significantly longer than that of patients with stage III/IV disease (*P* = 0.007), demonstrating a clear survival benefit with early diagnosis (Figure 1A). Pathogenic mutations that diminish FH enzymatic activity are mainly due to alterations in 2 sites: the catalytic active site and the inter-/intra-subunit interface sites (35, 36). Among the 41 detected missense mutations, 6 were in the active site, 7 were near the active site (within 10 Å from the active site), and 20 were located on the inter-/intra-subunit interface (Supplemental Figure 2B and Supplemental Figure 3). For missense mutations not previously reported as explicitly deleterious, the enzymatic activity was determined in cells that were compared with cells with either WT FH or the FH hotspot mutation R233L. Different *FH* cDNAs were constructed and expressed in FH-ablated (*Fh1^Δ/Δ^*) mouse kidney cells. Of these, N154H, R160S, V156I, G82R, T474P, K414E, C333Y, and R233L were detected by immunoblotting (Figure 1B), whereas N415I, L207R, G280V, and M382V were not, suggesting that the latter missense mutations are either poorly expressed or encode unstable proteins. The FH activity assays of the stable mutants showed that 8 of 9 proteins lost over 80% of their normal activity, whereas the N154H mutant lost approximately 40% activity (Figure 1C). Nevertheless, the patient bearing the N154H mutation (PID332) demonstrated an aggressive metastatic tumor with strong 2SC and negative FH IHC staining, which confirmed FH loss in the tumor (Figure 1D). Among all mutant cells, we found that intracellular fumarate levels were negatively correlated with enzyme activity (Figure 1E). Pathological examination of the FH-deficient RCC samples in our cohort revealed early-stage metastatic lesions. This is in line with previous work that demonstrated a higher metastatic capacity of FH-deficient cells mediated by fumarate-induced epithelial-mesenchymal transition (EMT) through the inhibition of the DNA demethylase TET2 (25). In line with that finding, G82R-, T474P-, and V156I-expressing cells exhibited a high migratory capacity through Matrigel, whereas N154H- and R160S-expressing cells showed a lower migratory ability (Figure 1F), and we observed a negative correlation between FH activity and migratory ability (Figure 1G).

### Untargeted plasma metabolomics analysis identified potential circulating biomarkers for FH-deficient RCC.

**Figure 2:**
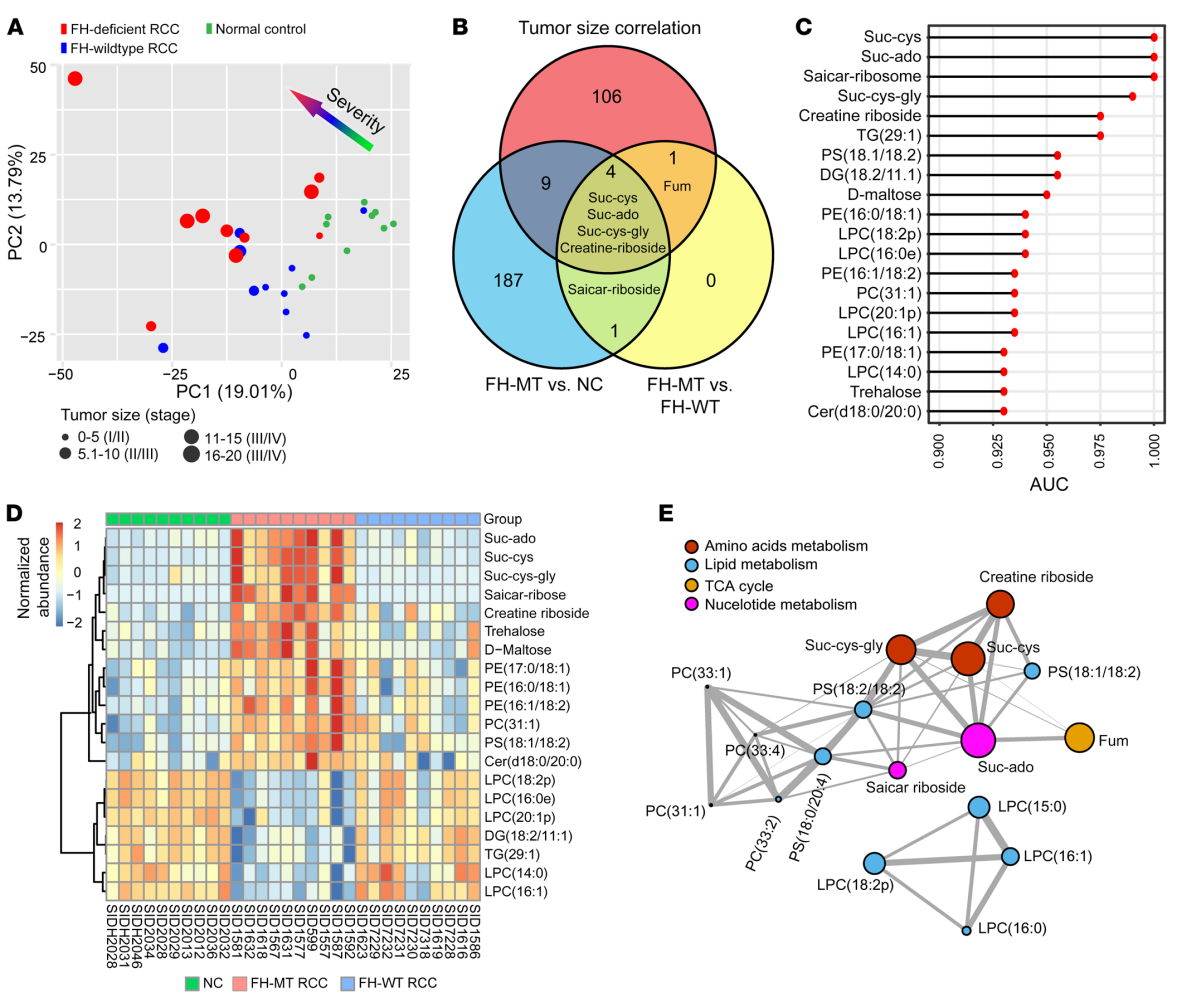
Identification of potential liquid biopsy biomarkers in RCC. (**A**) PCA of plasma samples (1,637 features) from NC individuals and patients with RCC categorized according to FH mutation status, tumor mass, and disease stage (represented by the size of each dot). (**B**) Venn diagram of altered plasma metabolites of FH-MT versus NC samples (blue) and FH-MT versus FH-WT samples (yellow) and tumor size correlation analysis (red). (**C**) The top 20 ROCAUC-ranked plasma metabolites discriminating FH-MT from FH-WT and NC samples. (**D**) Heatmap classification of FH-MT, FH-WT, and NC samples based on the metabolites in **C**. Scale bar: log_2_(normalized abundance). (**E**) Regularized partial correlation network of significantly altered metabolites in **B**. Each node represents a metabolite, and each edge represents the strength of the partial correlation coefficient between 2 compounds that were mapped into biochemical pathways. The size of each circle represents the strength of the correlation with tumor burden.

To perform extensive liquid biopsy metabolic profiling, we analyzed a total of 30 plasma samples as the discovery set, which included FH-MT RCC (*n* = 10), FH-WT RCC (*n* = 10), and NC (*n* = 10) samples (Supplemental Figure 1). We processed the samples using a standard protocol and analyzed them by untargeted metabolomics. After quality control and data filtering, we identified 1,637 metabolites, including 347 hydrophilic molecules and 1,290 hydrophobic molecules, across all samples. Principal component analysis (PCA) revealed potential clustering of plasma metabolites according to the FH genetic status in tumors and the stage of the disease (Figure 2A). To uncover the tumor-associated molecules, we generated volcano plots to compare the molecules in the FH-MT versus FH-WT RCC samples and FH-MT versus NC samples (FDR < 0.05) (Supplemental Figure 4, A and B, and Supplemental Table 2). We also performed tumor burden correlation analysis and identified 120 metabolites (FDR < 0.05) as important variables (Supplemental Figure 4C). Venn diagram analysis identified 4 metabolites that characterize FH deficiency–associated phenotypes that also correlate with tumor burden (Figure 2B). We carried out an independent ROCAUC analysis for all 1,637 metabolites by assigning mutated FH from the other 2 groups (Figure 2C). The top 20 metabolites from the ROCAUC analysis enabled clear separation of FH-MT samples from the other 2 groups (Figure 2D). Intriguingly, the top 5 AUC-ranked candidates highly overlapped with the 4 candidates identified from the Venn diagram analysis. These metabolites were suc-cys, suc-ado, succinic-cysteine-glycine (suc-cys-gly), and creatine-riboside (Figure 2, B and C). Although fumarate itself significantly distinguished FH-MT from FH-WT RCC and its plasma levels correlated with tumor burden (Figure 2B), because of its high variability and presence in NC samples, it was insufficiently sensitive to be used as a classifier (with ROCAUC = 0.86). In addition to the top ROCAUC-identified candidates elevated in FH-deficient patients, we also identified molecules with decreased levels in plasma; these molecules were mainly related to glycerol lipids and lysolipids. Furthermore, regulatory partial correlation networks were constructed to explore the biochemical functional relations between all significant metabolites (top 15 metabolites with a FDR < 0.05 in FH-MT versus NC and all 6 metabolites with a FDR < 0.05 in FH-MT versus FH-WT RCC taken from the volcano analyses) (Figure 2E). The topology of the network demonstrated that suc-cys, suc-cys-gly, and creatine-riboside were strongly correlated and appeared to be the best predictors of tumor burden, as indicated by the circle size in Figure 2E. Based on the above analysis, the plasma metabolic profiling revealed dramatic and programmed molecular changes in FH-MT RCC. In particular, suc-cys, suc-cys-gly, suc-ado, fumarate, and creatine-riboside were identified as potential biomarkers for monitoring the status and tumor burden of patients with FH variants.

### Validating the association between FH deficiency and plasma biomarkers in a preclinical RCC model.

**Figure 3:**
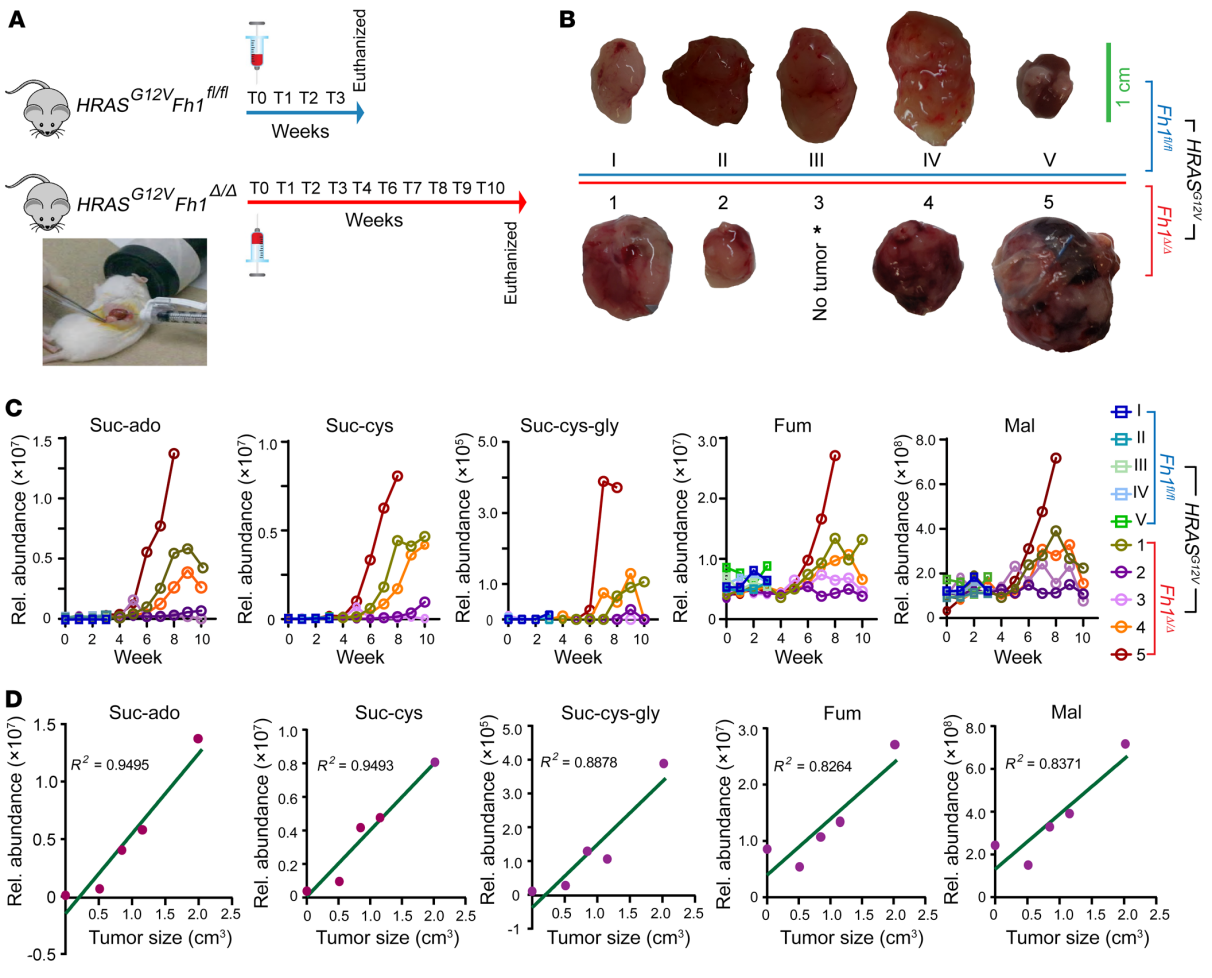
Association between FH deficiency and circulating biomarker levels in mice with CDX tumors. (**A** and **B**) Schematic overview of the generation of orthotopic kidney tumor xenografts by implantation of transformed epithelial kidney cells with the genotypes *HRAS^G12V^* *Fh1^Δ/Δ^* (*n* = 5 mice implanted) and *HRAS^G12V^* *Fh1^fl/fl^* (*n* = 5 mice implanted). Individual tumor sizes at the endpoints were documented. Scale bar: 1 cm. (**C**) Longitudinal monitoring of the indicated plasma metabolites in mice bearing either WT FH tumors (*HRAS^G12V^ Fh1^fl/fl^*, *n* = 5) or FH-deficient tumors (*HRAS^G12V^* *Fh1^Δ/Δ^*, *n* = 5). (**D**) Correlation between the maximal measured level of each metabolite during the course of the study and tumor volume (cm^3^) at the endpoint in each *HRAS^G12V^* *Fh1^Δ/Δ^*–engrafted mouse. Mal, malate; Rel., relative.

To better appreciate the kinetics of the potential plasma metabolic biomarkers for RCC, cell-derived tumor xenografts (CDXs) in mice were used to monitor the impact of tumor growth on plasma metabolites. Human *HRAS^G12V^* was overexpressed in genetically modified murine FH-expressing (*Fh1^fl/fl^*) and FH-deficient (*Fh1^Δ/Δ^*) epithelial kidney cells (37). *Fh1^fl/fl^ HRAS^G12V^* cells and *Fh1^Δ/Δ^ HRAS^G12V^* cells were orthotopically injected into the kidneys of 5-week-old immunosuppressed mice (Figure 3A). Since the FH-deficient cells proliferated more slowly, mice xenografted with these cells survived 8–10 weeks after injection, whereas those xenografted with WT cells died in the third week. Plasma metabolites were analyzed at baseline and once a week following transplantation. Tumor sizes at the endpoints ranged from 0.5 to 1.5 cm in diameter (Figure 3B). We did not detect creatine-riboside in plasma from the mice, regardless of FH status. However, suc-ado, suc-cys, and suc-cys-gly were specifically detected in the plasma of mice with FH-deficient CDXs and proportionally increased over time along with tumor progression (Figure 3C). These metabolites were detected in the plasma 3–4 weeks prior to the endpoint, indicative of their potential to diagnose FH deficiency early. More intriguingly, the maximal plasma levels of these metabolites were proportional to the tumor mass at the endpoint (Figure 3D).

**Figure 4:**
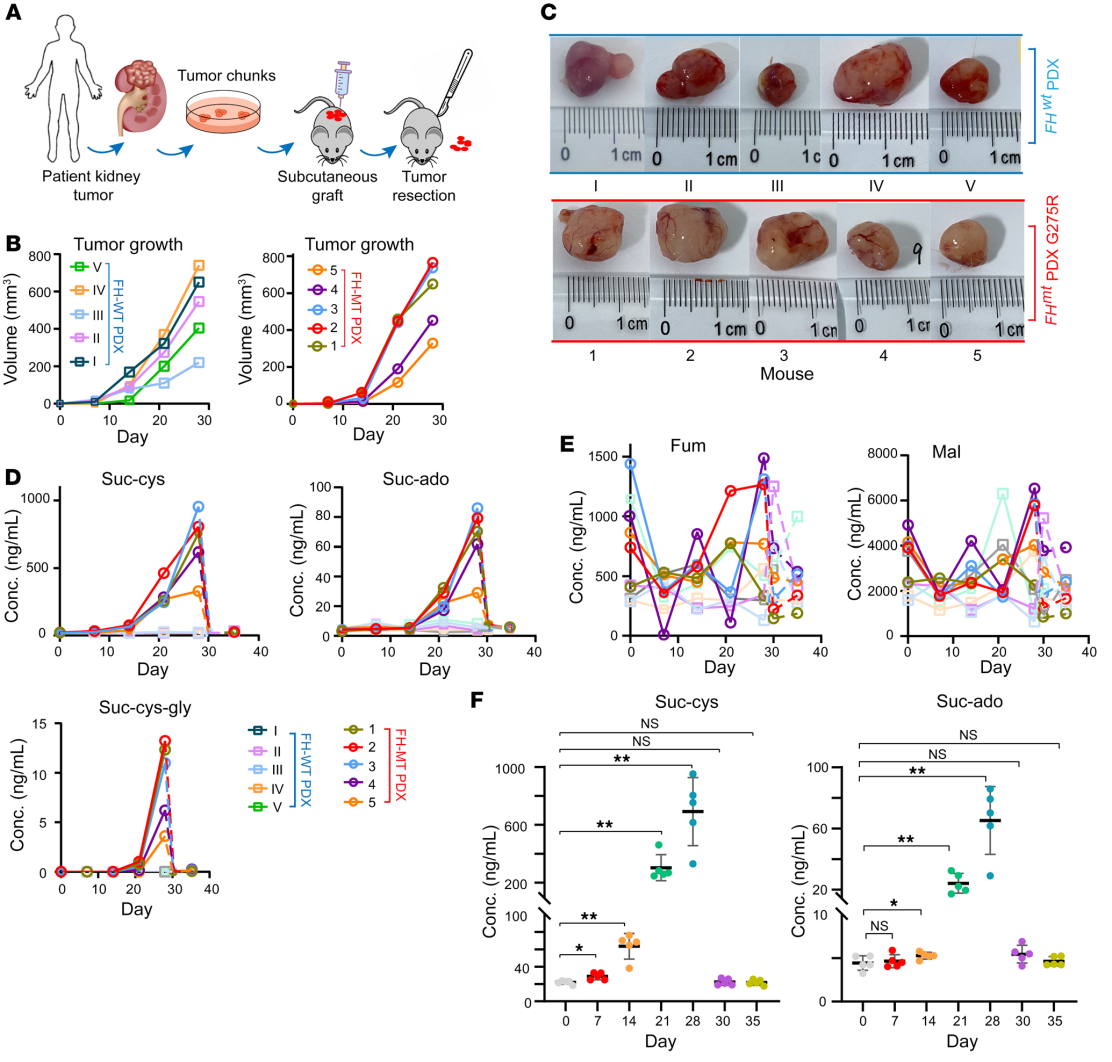
Relation between FH-MT and plasma biomarker levels in mice with PDX tumors. (**A**) Schematic overview of the generation of subcutaneous xenografts by transplantation of human kidney tumors with the genotypes FH-WT ccRCC (*n* = 5 mice engrafted) or FH-MT RCC (*n* = 5 mice engrafted). Surgical resection of the subcutaneous FH-MT PDX was performed upon reaching the endpoint, and mice were sacrificed 1 week after the resection surgery. (**B**) Growth rate of each PDX tumor of the indicated genotype. (**C**) Representative images of individual tumors at the endpoints. (**D** and **E**) Longitudinal monitoring of the indicated plasma metabolites in mice bearing a PDX of either a FH-WT ccRCC tumor (*n* = 5 mice) or a FH-deficient tumor (*n* = 5 mice). (**F**) Comparisons of plasma metabolites over time in mice engrafted with FH-MT tumors (*n* = 5). All data are presented as the mean ± SEM. *P < 0.05 and **P < 0.01, by paired, 2-tailed Student’s *t* test, with *P* value–adjusted Bonferroni correction. Conc., concentration.

Patient-derived tumor xenografts (PDXs) were generated to monitor the effects of a genuine human tumor on plasma metabolites (Figure 4A). One PDX originated from a left renal mass from a male patient with FH-MT, who was diagnosed with a germline alteration of FH c.823G>A (p.G275R), whereas the FH-WT control tumor was from a right kidney lesion from a patient with ccRCC. Both PDXs were subcutaneously transplanted into immunodeficient mice. The FH-MT tumor grew at a rate comparable to the that of the WT tumors (Figure 4B and Supplemental Figure 5A), and the tumor size at the endpoints ranged from 1.0–1.5 cm in diameter (Figure 4C). We performed surgical resection upon reaching the endpoint to clinically imitate nephrectomy, and mice were sacrificed 1 week after the surgery (Supplemental Figure 5A). We analyzed plasma at baseline and weekly following transplantation as well as on day 1 and day 7 after tumor resection. IHC of PDX samples revealed strong 2SC and negative FH staining in FH-MT PDXs (Supplemental Figure 5B). As with CDXs, suc-ado, suc-cys, and suc-cys-gly were particularly detected in the mice with FH-deficient PDXs and proportionally increased over time along with tumor growth (Figure 4, D and E). Notably, elevated plasma suc-cys levels were detected 7 days after transplantation when the tumor mass ranged between 2 and 3 mm in diameter (*P* = 0.0195, Figure 4F). Suc-ado levels appeared to increase in the plasma on day 14 after transplantation when tumors grew to 5–6 mm in size (*P* = 0.0301, Figure 4F). These results demonstrated that suc-cys and suc-ado allow the detection of small-mass tumnors and have excellent sensitivity to determine the status of tumors. Importantly, plasma metabolites fell to basal levels 1 day after surgical resection of the subcutaneous tumor.

**Figure 5:**
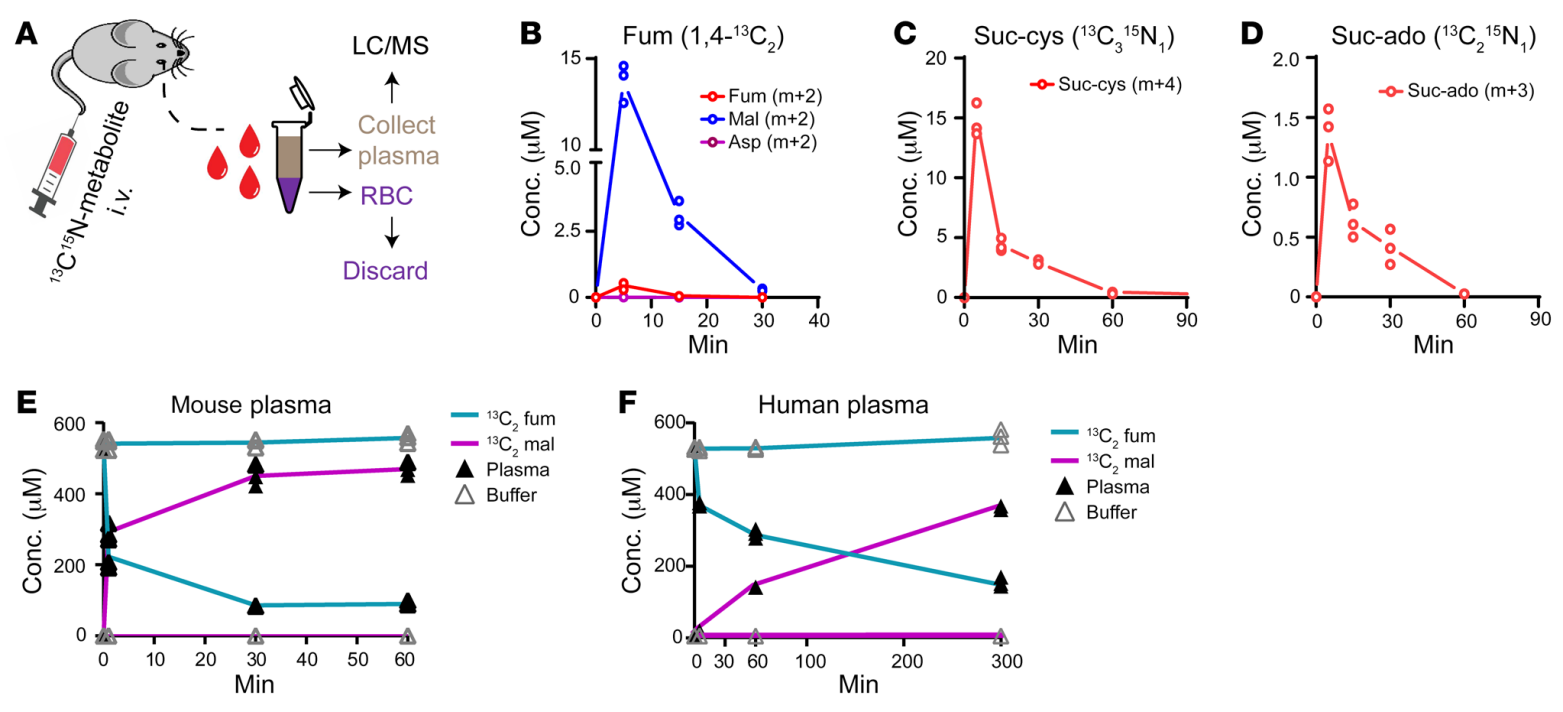
The biochemical conversion of plasma molecules in mice. (**A**) Schematic representation of the in vivo metabolic conversion of labeled intravenously injected metabolites in mice. LC/MS, liquid chromatography/mass spectrometry. (**B**–**D**) Mice were injected with 1,4-^13^C_2_-fumarate (**B**), ^13^C_3_^15^N_1_ suc-cys (**C**), or ^13^C_2_^15^N_1_ suc-ado (**D**), and the injected metabolic tracer as well as its metabolic products were analyzed. (**E** and **F**) In vitro metabolic conversion of fumarate into malate in mouse plasma (**E**) or human plasma (**F**) incubated with 1,4-^13^C_2_-fumarate for the indicated duration. In each assay, the buffer was used as a nonenzyme control. All experiments were performed independently 3 times. All data are presented as the mean ± SEM.

Similar to the findings of the discovery study in patients, fumarate itself did not correlate well with the kinetics of tumor growth or the tumor mass at the endpoint in either xenograft model (Figure 3, C and D, Figure 4E). Surprisingly, malate exhibited a pattern rather similar to that of fumarate in the plasma (Figure 3, C and D, and Figure 4E). These unexpected results suggest that fumarate may be converted to malate as a result of FH activity in plasma. A bolus of [1,4]-^13^C_2_–fumarate was injected intravenously (2 mg/kg) and traced into malate (Figure 5A). ^13^C_2_-fumarate was barely detected in the plasma of mice even 5 minutes after injection. However, ^13^C_2_-malate was readily detected, peaking at 5 minutes, as were smaller amounts of ^13^C_2_-aspartate (Figure 5B). Such conversion also took place in mouse and human plasma in vitro (Figure 5, E and F). Of note, no cofactors are required for this hydration reaction. In contrast to fumarate, ^13^C^15^N isotope–labeled suc-cys and suc-ado were metabolically stable during circulation, with a half-life of 20–30 minutes without further conversion to other metabolites such as adenosine, cysteine, or succinate (the building blocks for these molecules) (Figure 5, C and D). Together with the low (barely detected) basal levels of suc-cys and suc-ado in normal plasma, these results support the use of suc-cys and suc-ado, but not fumarate, as plasma biomarkers for reporting the presence of FH-deficient tumors.

### Circulating suc-cys is an enzymatic product of fumarate-derived metabolism.

**Figure 6:**
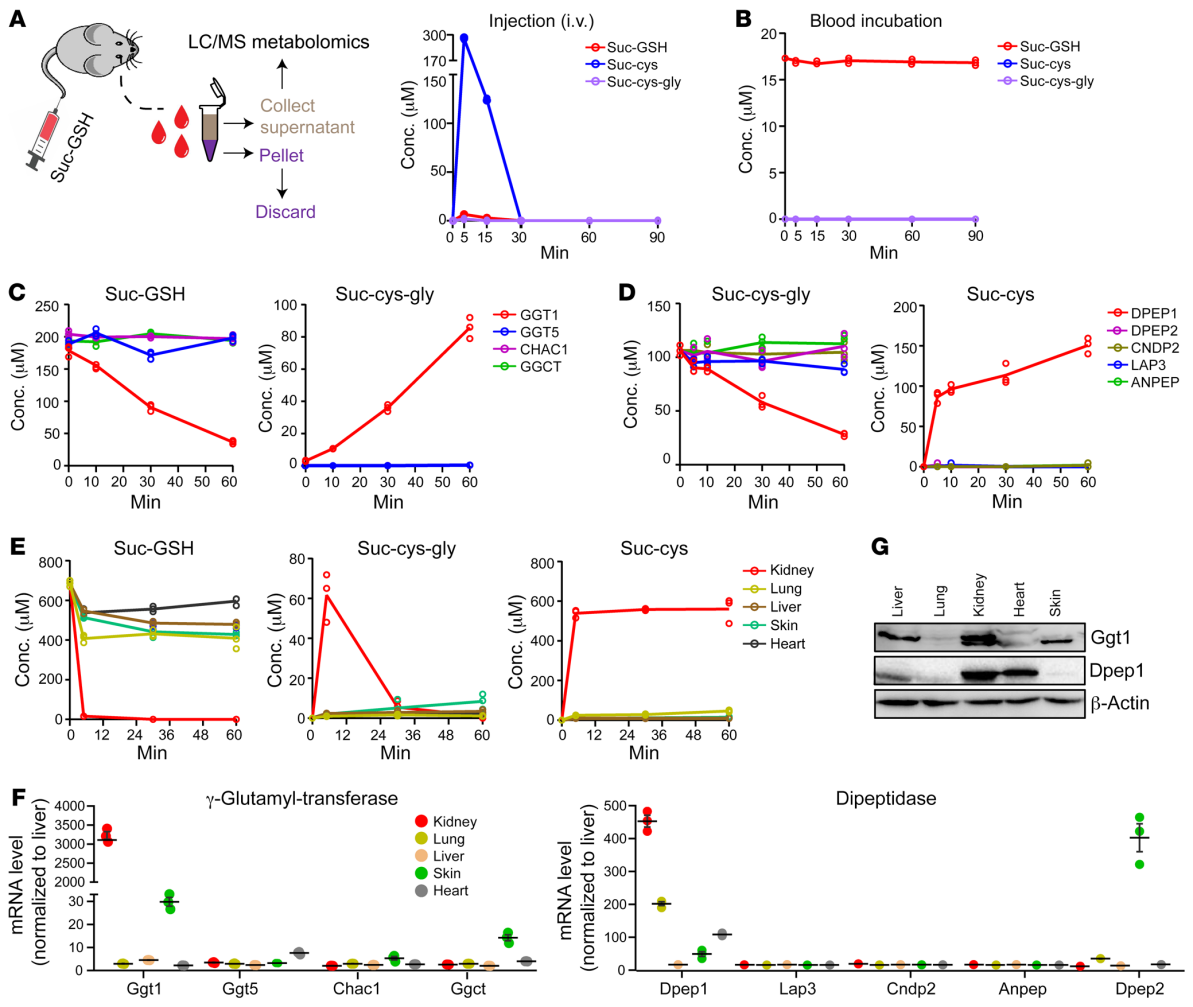
Circulating suc-cys is a product of the enzymatic cascade of the GGT1-DPEP1 axis. (**A**) Schematic representation of intravenous injection of suc-GSH into mice and the metabolic fate in vivo. (**B**) Analysis of the in vitro assay of suc-GSH in mouse plasma. (**C**) Analysis of the in vitro enzymatic conversion of suc-GSH into suc-cys-gly by the recombinant proteins. (**D**) Analysis of the in vitro enzymatic conversion of suc-cys-gly into suc-cys by the recombinant human proteins. (**E**) Assessment of the ability of the indicated mouse tissue homogenates to catabolize the conversion of suc-GSH into suc-cys-gly and suc-cys. (**F**) mRNA levels of different transpeptidases and dipeptidases in various mouse tissues (normalized to liver) were determined by quantitative PCR (qPCR). (**G**) Immunoblotting was used to measure GGT1 and DPEP1 expression. All experiments were performed independently 3 times. All data are presented as the mean ± SEM.

Suc-ado accumulates in FH-deficient cells due to dysregulated purine biosynthesis associated with ADSL inhibition by fumarate (38). However, the source of circulating suc-cys is unknown. We and others previously studied the accumulation of succinic-GSH (suc-GSH) in human and mouse tissues from FH-deficient tumors (26, 39). However, suc-GSH was not detected in the plasma of patients or mice bearing FH-deficient CDXs (not shown). This indicates that suc-GSH may be catabolized to suc-cys-gly and further to suc-cys in cells or in the blood. To test this hypothesis, suc-GSH was injected intravenously (5 mg/kg) into mice, and plasma was collected after the injection (Figure 6A). Strikingly, we detected approximately 300 μM suc-cys within 5 minutes of injection of suc-GSH, whereas we detected only approximately 5 μM suc-GSH and approximately 2.0 μM suc-cys-gly (Figure 6A). This finding strongly implies a catalytic conversion of suc-GSH to suc-cys (likely via suc-cys-gly) in the plasma. However, when incubated with mouse plasma in vitro, suc-GSH was converted to neither suc-cys-gly nor suc-cys (Figure 6B). These results suggest that suc-GSH cannot be catabolized spontaneously; rather, the enzymes responsible for its catabolism may exist within a tissue/local organ environment but not freely in the plasma. Suc-GSH has a structure similar to that of GSH; hence, recombinant transpeptidases, i.e., γ-glutamyl transpeptidase (GGT), glutathione-γ glutamyl cyclotransferase (CHAC1) and γ-glutamyl cyclotransferase (GGCT) were constructed and tested (see Methods and Supplemental Figure 6, A and B). Of the recombinant proteins, only GGT1 was shown to catabolize suc-GSH, generating nearly equimolar amounts of suc-cys-gly, but there was no further production of suc-cys (Figure 6C). Suc-cys-gly is a dipeptide derivative that may be recognized by various peptidases, including alanyl aminopeptidases (ANPEPs) and leucine aminopeptidases (LAPs), cytosolic nonspecific dipeptidases (CNDPs), and membrane-bound dipeptidases (DPEPs). We therefore produced recombinant active ANPEPs, LAP3, CNDP2, DPEP1, and DPEP2 (see Methods, Supplemental Figure 6C and Supplemental Figure 7). The enzymatic assay showed that, of these enzymes, only DPEP1 enabled the conversion of GGT1-generated suc-cys-gly into equimolar amounts of suc-cys (Figure 6D).

**Figure 7:**
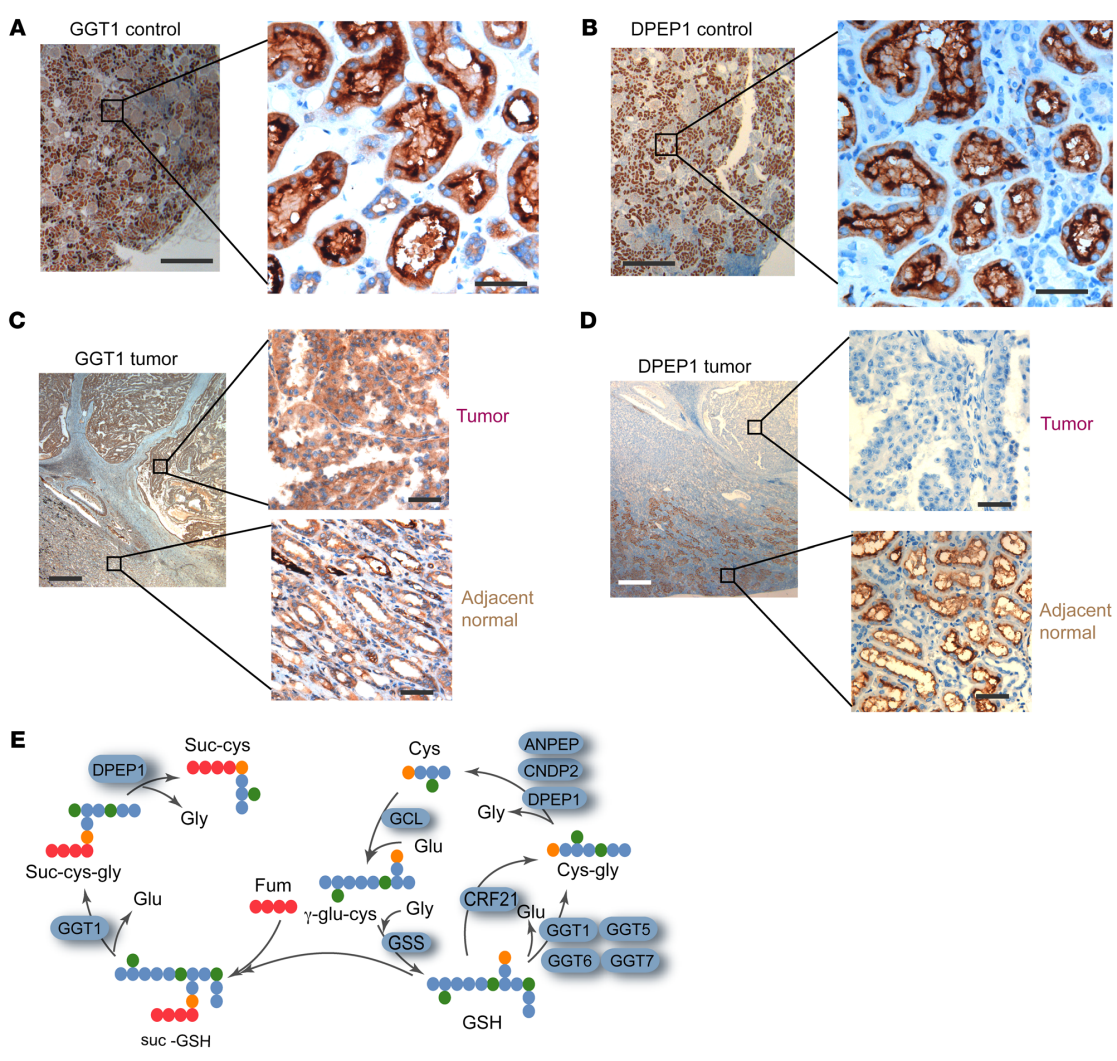
Plasma suc-cys is generated by GGT1-DPEP1 cooperativity in the kidney. (**A** and **B**) Representative IHC staining for GGT1 (**A**) and DPEP1 (**B**) in healthy human kidney tissue showing strong plasma membrane and cytoplasmic positivity in renal tubular cells. (**C** and **D**) Representative IHC staining for GGT1 (**C**) and DPEP1 (**D**) in FH-deficient kidney tumor tissue and adjacent normal tissue from 1 patient. All experiments were performed independently 3 times. (**E**) A workflow of suc-cys generation from suc-GSH via peptidases. Scale bars: 800 μm (left) and 50 μm (right) (images in **A**–**D**).

Since the catabolism of suc-GSH took place in the circulation in vivo but not in plasma in vitro, we tested whether tissue-associated enzymes are responsible for suc-GSH catabolism. Tissue homogenates from the liver, kidney, lung, heart, and skin were used as sources for suc-GSH-catabolizing enzymes. The assays clearly showed that, of the tested tissue homogenates, only the kidney homogenate was able to metabolize suc-GSH to suc-cys-gly and further to suc-cys (Figure 6E). Intriguingly, both GGT1 and DPEP1 are highly expressed in the kidney, and both are primarily located on the membrane of tubule cells (Figure 6, F and G, and Figure 7, A and B). IHC analysis of FH-deficient RCC showed that GGT1 was expressed in the tumor cells, whereas DPEP1 was primarily confined to the normal adjacent tissues (Figure 7, C and D). Together, these results demonstrated that an organized enzymatic cascade and cooperativity within the tumor microenvironment resulted in the conversion of tumor-derived suc-GSH into circulating suc-cys (Figure 7E and Supplemental Figure 8).

### Suc-ado and suc-cys are sensitive and specific metabolic biomarkers that accurately reflect the progression of FH-deficient RCC.

**Figure 8:**
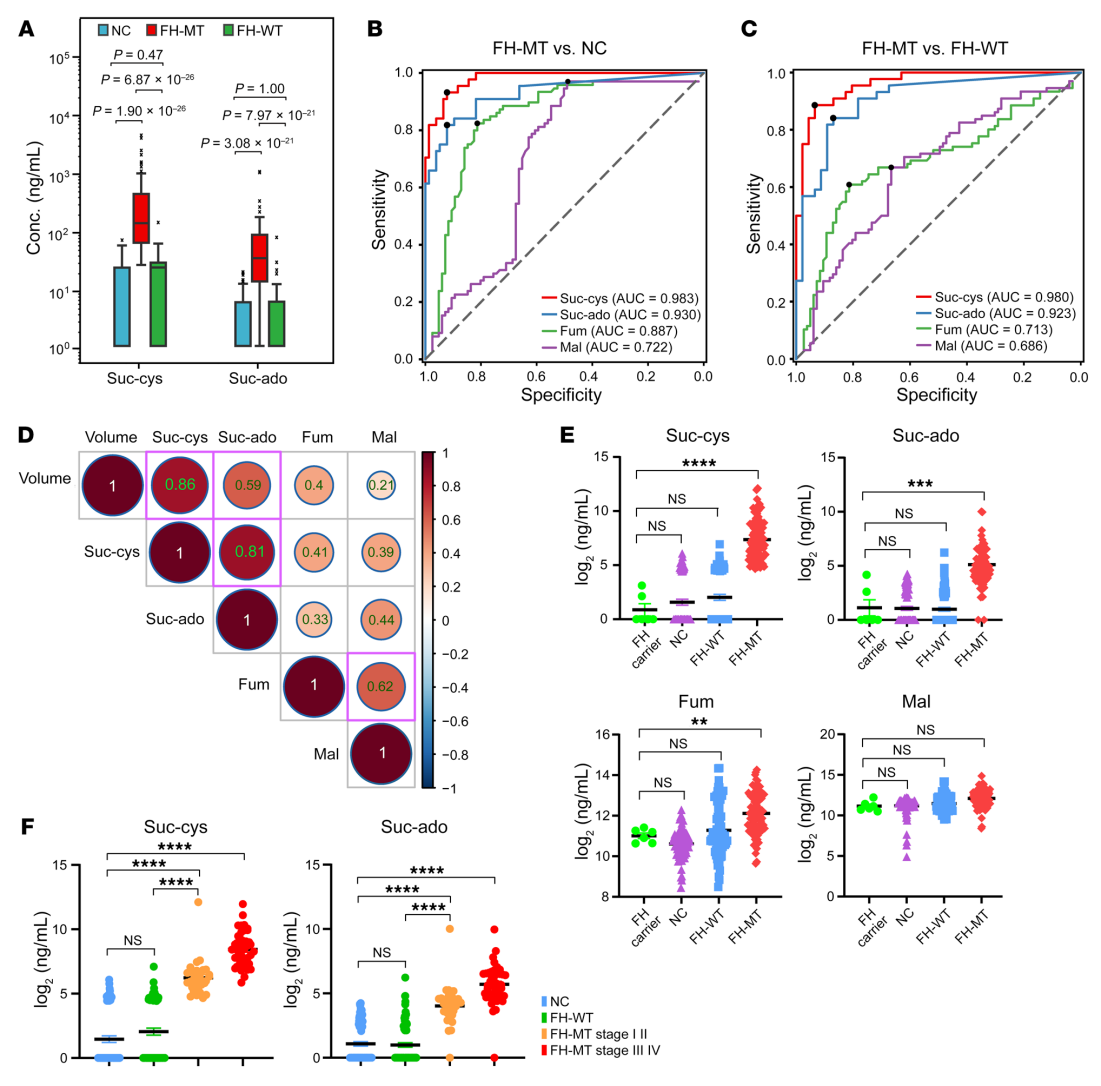
Circulating suc-ado and suc-cys levels can be used to accurately identify FH-deficient RCC. (**A**) Box plot analysis showing the distribution of plasma metabolites across NC, FH-MT, and FH-WT. Wilcoxon rank-sum test *P* values with Bonferroni correction were calculated. (**B** and **C**) Logistic regression ROCAUC analyses of metabolites in FH-MT versus NC samples (**B**) and FH-MT versus FH-WT samples (**C**). (**D**) Spearman’s correlation coefficient between tumor volume (log_2_ mm^3^) and potential plasma biomarker levels (log_2_ ng/mL). (**E**) Scatter plot analysis showing the distribution of the levels of plasma metabolites between FH-MT carriers (no tumor), NCs, and patients with FH-WT or FH-MT RCC. All data are presented as the mean ± SEM. **P < 0.01, ***P < 0.001, and ****P < 0.0001, by unpaired, 2-tailed Student’s *t* test, with *P* value–adjusted Bonferroni correction. (**F**) Scatter plot analysis showing the distribution of the levels of plasma metabolites across NCs and patients with FH-WT, FH-MT stage I/II, or FH-MT stage III/IV. All data are presented as the mean ± SEM. ****P < 0.0001, by unpaired, 2-tailed Student’s *t* test, with *P* value–adjusted Bonferroni correction.

To validate observations made with the discovery set and the mouse preclinical models, we analyzed a larger separate validation set of 238 plasma samples from 67 patients with FH-MT, 78 patients with FH-WT, and 67 NC individuals (Supplemental Figure 1). The absolute plasma concentrations of suc-ado and suc-cys were quantified using ^15^N-^13^C isotope–labeled molecules synthesized in-house as internal references. Both metabolites were significantly elevated in the plasma of the FH-MT group (Figure 8A and Supplemental Table 3). Suc-cys distinguished FH-MT RCC from NC samples with a ROCAUC of 0.983 (sensitivity = 0.928, specificity = 0.935, cutoff = 32 ng/mL), and suc-ado had a ROCAUC of 0.930 (sensitivity = 0.831, specificity = 0.922, cutoff = 12 ng/mL, Figure 8B). The biomarkers could also distinguish FH-MT from FH-WT RCC (suc-cys: ROCAUC = 0.980; suc-ado: ROCAUC = 0.923, Figure 8C), suggesting that these biomarkers can be used for both routine screening of carriers of the germline FH mutant (the prevalence of FH mutations in the population is estimated to be 1 in 1,000) (40) and to perform differential diagnosis of patients with RCC.

In contrast to suc-cys and suc-ado, and similarly to the discovery set and the preclinical study, fumarate and malate were not sufficiently suitable classifiers, even in the large validation set (Figure 8, B and C). Furthermore, the positive correlation between malate and fumarate levels supports the notion that fumarate is converted to malate in the plasma (Figure 8D). A strong correlation between suc-cys and suc-ado was also demonstrated, and each of these metabolites strongly correlated with tumor volume (Figure 8D). In contrast, fumarate and malate correlated poorly with tumor size (Figure 8D).

Given that FH is a homotetramer, it is plausible that missense germline mutations may affect FH functions, even in a heterozygous state. Therefore, we compared plasma suc-cys, suc-ado, fumarate, and malate levels between FH-MT carriers with 1 mutant allele (but no tumors) with all other groups. No significant elevation of any of these metabolites was detected in the plasma of the FH carrier group in comparison with the control NC group (no FH carriers) or the FH-WT RCC samples, supporting a lack of a dominant negative effect on patients’ plasma metabolites (Figure 8E). To better appreciate the sensitivity and diagnostic capability of suc-cys and suc-ado, we quantified the plasma levels of these biomarkers in patients with early stages (I and II) and late stages (III and IV) of the disease. We found that both metabolites were significantly elevated in stage I–II tumors compared with NC individuals (*P* < 0.0001) or patients with FH-WT (*P* < 0.0001), suggesting that these circulating biomarkers make it possible to diagnose early FH-MT tumor (Figure 8F).

**Figure 9:**
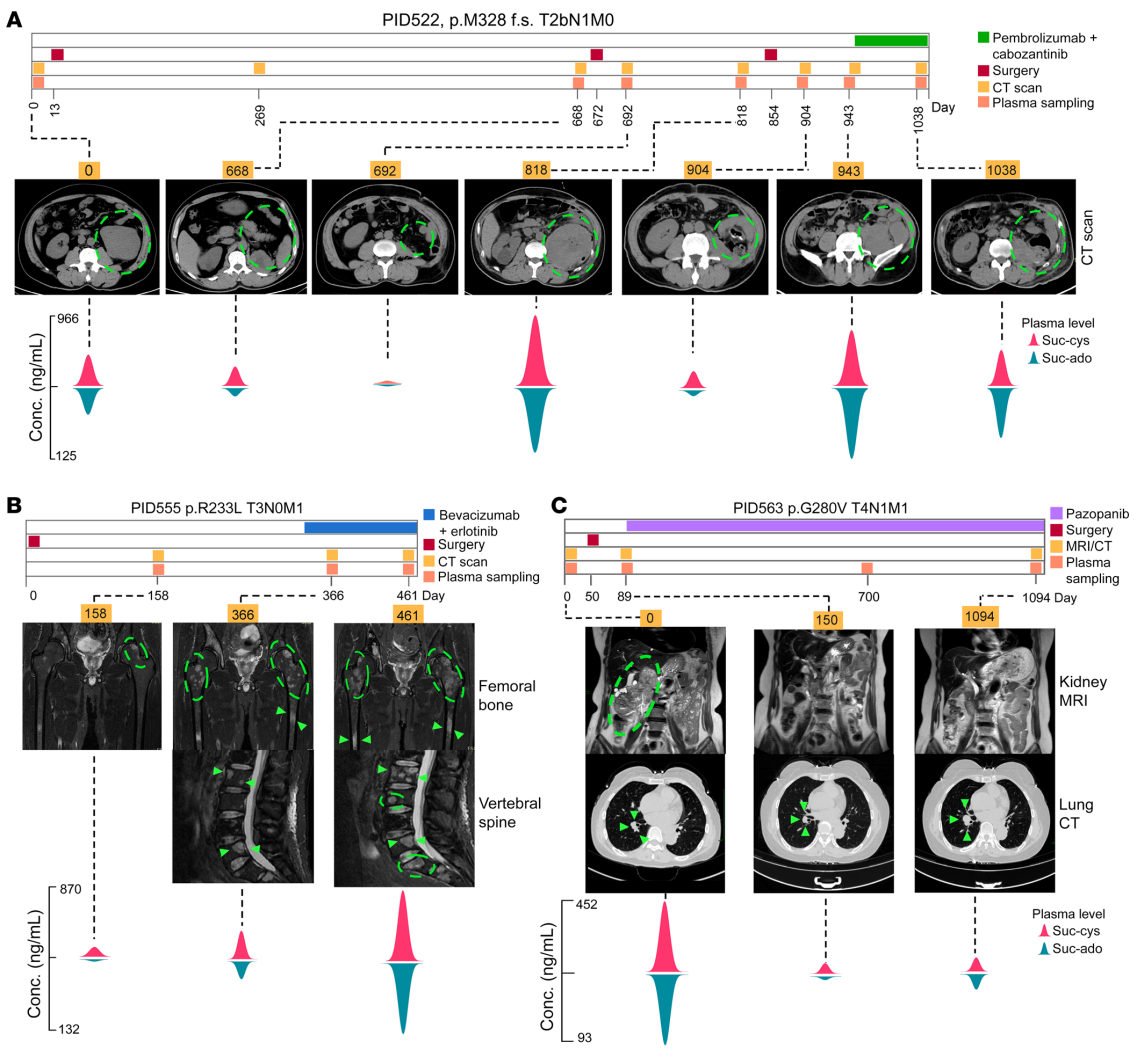
Plasma suc-ado and suc-cys levels can be used to accurately monitor tumor progression. (**A**–**C**) Longitudinal monitoring of the dynamics of plasma suc-ado and suc-cys during multiple clinical events in 3 patients: PID522 (**A**), PID555 (**B**), and PID563 (**C**). In each panel, the top row shows the distinct time of each clinical event, the middle row shows examples of medical imaging at the indicated clinical events, and the lower row shows the “wave plot,” the height of which represents the concentration of plasma metabolites at the indicated time point.

The surveillance and monitoring utility of these molecules was tested by longitudinally tracking the levels of circulating biomarkers in 5 patients at multiple time points, i.e., at the initial diagnosis, surgery, recurrence, and throughout systemic therapy (Figure 9, A–C, and Supplemental Figure 9, A and B). (a) Patient PID522 had a left kidney lesion (10 cm) at the initial diagnosis with plasma suc-cys and suc-ado levels of 419.54 and 61.21 ng/mL, respectively, well beyond the diagnostic cutoff (Figure 9A). On day 668, the plasma levels of suc-cys and suc-ado had increased, indicating disease recurrence, which was confirmed by a CT scan. A partial nephrectomy was performed on day 672, and the plasma levels (day 692) immediately dropped to undetectable levels. Regrettably, the plasma suc-cys and suc-ado levels were elevated by day 904, which was 50 days after the third resection (day 854), and continued to rise, doubling to 483.78 and 94.70 ng/mL, respectively, in merely 39 days (day 943). We thus suspected the presence of distant metastases, which was confirmed by PET-CT, and we initiated systemic therapy. The declining plasma levels indicated an effective response after 3 months of treatment with pembrolizumab in combination with cabozantinib, and a shrinking tumor was confirmed by CT. (b) Patient PID555 developed metastases in the spine and femoral bone 158 days after surgery (Figure 9B). The plasma levels concurrently increased, along with uncontrolled expansion of bone metastases within 3 months (days 366 and 461) because of the treatment’s poor efficacy. (c) Patient PID563 was initially diagnosed with an advanced localized lesion and underwent surgery for a kidney tumor on day 50 (Figure 9C). We started therapy with pazopanib early, 39 days after surgery, and the plasma biomarkers reliably reflected the presence of a small (1–1.5 cm) lung metastatic lesion that remained stable for 3 years. (d) Patient PIDN7326 had brain and bone metastases with plasma suc-cys and suc-ado levels of 1,112.29 and 170.48 ng/mL, respectively (Supplemental Figure 9A). Bevacizumab, in combination with erlotinib, was given (day 56) for 3 months. However, the patient did not achieve a response and exhibited progressive disease (days 120 and 143). A second line of therapy with pembrolizumab plus cabozantinib was introduced, and 40 days later, the tumor began to shrink (day 209). We maintained this treatment for 14 months, and the patient showed a partial response, which was confirmed by the lower biomarker values. (e) Patient PID7217 had a right kidney mass at the initial diagnosis and developed metastases to the bone, liver, and lungs (Supplemental Figure 9B). The levels of suc-cys and suc-ado were as high as 339.28 ng/mL and 49.51 ng/mL, respectively (day 0). The patient was treated with pazopanib in combination with nivolumab but showed no response (day 91). We thus treated the patient with bevacizumab plus erlotinib for 8 months, the yet the patient’s plasma levels of suc-cys and suc-ado remained high at day 166 (1,248.46 ng/mL and 115.63 ng/mL, respectively) and day 357. Therefore, we changed the therapy to cabozantinib in combination with pembrolizumab, and, by day 462, the overall tumor mass had substantially shrunk, as had the biomarkers levels (suc-cys = 13.91 ng/mL and suc-ado = 4.38 ng/mL). Stable disease was maintained by this strategy for 8 months (Supplemental Figure 9B). The above longitudinal studies demonstrated that the dynamic concentrations of suc-cys and suc-ado in the circulation were proportional to tumor mass, regardless of local or metastatic lesions. At multiple clinical time points, including the initial diagnosis, disease recurrence, surgery, and systemic therapy, these circulating biomarkers allowed us to monitor FH-MT tumor status.

## Discussion

FH-deficient neoplasms are extremely aggressive tumors. Primary knowledge of the FH status in tumors dictates surgical strategies, and early-stage detection improves clinical outcomes (7–10). Forde et al. reported a cohort comprising 23 patients with RCC among 185 FH-deficient individuals from 69 families (7). The mean survival of patients with stage III/IV RCC (*n* = 9) was significantly shorter (15.8 months) than that of patients with stage I/II RCC (80.7 months), and all patients with stage III/IV RCC died within 36 months of their diagnosis. Chen et al. reported a study of an uncharacterized RCC cohort, in which FH-deficient patients with stage III RCC(*n* = 4) displayed the worst progression-free survival (PFS) and overall survival (OS), and all died within 40 months of their diagnosis (9). Although early detection will not alter the nature of highly metastatic disease, improving diagnosis can increase the chances of detecting the disease at an early stage, at which point aggressive surgery will improve the clinical outcome.

FH-deficient neoplasms were thought to have a low incidence but high mortality rate. However, the prevalence of deleterious FH mutations in the population might be significantly higher than previously assumed. Based on population-based genomic data, Shuch et al. found that 1 in 1,000 individuals carries a FH alteration, and the lifetime kidney cancer risk for mutation carriers is estimated to be 5%–17% (40). However, to our knowledge, no liquid biopsy or other noninvasive biomarker exists for the early diagnosis of FH-deficient RCC. This study revealed metabolically stable circulating metabolites to be sensitive and specific predictors of FH deficiency, tumor burden, and tumor stage. These accurate, cost-effective, and easily implementable biomarkers can be used to ultimately reduce the morbidity and mortality of patients with FH-deficient RCC.

In this study, we built a large RCC cohort with individuals from multiple centers, including 93 plasma samples derived from 77 patients with various stages of FH-MT RCC. The sample collection and monitoring spanned from 2016 to 2021. Using plasma metabolomics analysis, we identified suc-cys and suc-ado as circulating biomarkers of FH-deficient tumors. Their plasma concentrations reflected the biallelic inactivation of *FH* and correlated with tumor burden. We consistently found suc-cys and suc-ado levels to be useful for multiple aspects of disease management, including screening, diagnosis, and determining whether surgery and therapy would be effective. Therefore, the monitoring of plasma suc-cys and suc-ado levels is a noninvasive, rapid, accurate, and cost-effective strategy that complements molecular genetic analyses and repeated CT and MRI scans. Of note, recurrent FH-deficient tumors exhibit extremely rapid growth and can double in mass within a few days or months (Figure 9, A and B). Therefore, very frequent monitoring is required for early detection of the disease and for providing the best intervention window. The cost and health risks of CT and MRI (e.g., severe nephropathy), which include x-ray exposure and/or contrast agent exposure, prohibit their frequent use. Furthermore, imaging inaccuracies such as pseudoprogression mediated by therapy-induced necrosis are a challenge (41). Thus, determining the levels of our effective plasma biomarkers is, we believe, an excellent complementary strategy for frequent monitoring.

Here, we found that GSH succination is a major source of suc-cys, as suc-GSH accumulates in FH-deficient tumors and is then metabolized by GGT (e.g., GGT1) and renal DPEPs (e.g., DPEP1), which are largely present on the membrane and in the cytoplasm of epithelial cells in renal tubules. In line with that, we detected significant elevation in the expression of genes associated with GSH precursor biosynthesis in FH-deficient tumors, and we found the GGT1 and DPEP1 proteins in the vicinity of the tumors. Altogether, our data demonstrated how disrupted metabolism caused by FH deficiency can lead to unique metabolic traits that produce stable circulating metabolites that can serve as sensitive and specific biomarkers for the early diagnosis and prognostication of FH-MT RCC.

## Methods

### Clinical study

#### Study cohorts.

Eighty-eight patients with FH-WT RCC were newly recruited from 3 centers and had the following RCC subtypes: ccRCC (*n* = 26), pRCC Type 1 (*n* = 21), pRCC type 2 (*n* = 33), Xp11.2 translocation RCC (*n* = 2), and uncharacterized pRCC (*n* = 6). IHC and DNA genetic sequencing were performed to confirm the WT FH. Seventy-seven patients with FH-deficient (FH-MT) RCC from 15 centers were selected from our established cohort (34). Of the 77 total patients, 70 had tumors with germline FH mutations and 7 had sporadic tumors with somatic FH mutations, spanning all tumor stages. Their disease was confirmed by IHC and DNA genetic sequencing. Finally, 87 age- and sex-matched healthy individuals were recruited as the no-tumor controls (NC). Collectively, 252 individuals from 16 centers comprised the SHJTUCMRJ cohort (Supplemental Figure 1 and Supplemental Figure 2A).

#### Sample inclusion.

Blood samples were collected from participants from each center. For sample collection, no specific timing (e.g., morning or afternoon) was required, nor were the participants asked to fast or restrict their activities, alcohol consumption, etc. Hemolysis was the quality control criterion for the exclusion of samples. Two of 270 samples were excluded because of hemolysis. Ultimately, a total of 268 human plasma samples were included in the study.

#### Study design.

A total of 93 samples from 77 patients were included in the FH-deficient (FH-MT) RCC group. Ten samples from 10 patients, covering disease stages I, II, III, and IV at the time of collection, were selected as the discovery set. A separate set of 83 samples from 67 patients was selected as the validation set. A total of 88 samples were selected for the FH-WT group. Also, 10 samples from 10 patients, covering stages I, II, III, and IV of FH-WT RCC at the time of sample collection, were selected as the discovery set. A separate set of 78 samples from 78 patients covering different disease stages was selected as the validation set. A total of 87 samples were selected for the NC (no tumor) group. Ten samples from different individuals were randomly selected as the discovery set, and a separate set of 77 samples was selected as the validation set (Supplemental Figure 1).

#### Clinical outcomes.

The patients were underwent regular assessments by a urologist (pathological examinations, radiologic examinations, and laboratory tests) every 6–10 weeks. The treatment response and outcome were defined according to Response Evaluation Criteria in Solid Tumors (RECIST) 1.1 guidelines. All imaging studies to assess tumor burden of the local lesion or of metastatic lesions for all patients were performed by a single radiologist, who applied the RECIST 1.1 criteria.

### Cell lines

*Fh1*-deficient clone (*Fh1^–/– CL19^*) and *Fh1*-proficient cells (*Fh1^fl/fl^*) were from murine epithelial cells taken from the kidney as previously described (37). All cell lines were cultured in DMEM (Hyclone, catalog SH30081) supplemented with 10% heat-inactivated serum (Gibco, Thermo Fisher Scientific, catalog 16170078), 2 mM glutamine (Gibco, Thermo Fisher Scientific, catalog 25030149), and 1 mM pyruvate (Gibco, Thermo Fisher Scientific, catalog 11360070) and were regularly tested using a mycoplasma detection kit (R&D Systems, catalog CUL001B) to ensure they were mycoplasma free.

### Tumor xenograft models

Immunodeficient NOD-SCID γ (NSG) mice were obtained from the Pre-Clinical Research Authority (PCRA) inbred colony kept in the animal facility at Technion. *Fh1^fl/fl^* and *Fh1^Δ/Δ^* cells were transformed by a pWZL retroviral vector encoding human oncogenic *HRAS^G12V^* to generate *Fh1^fl/fl^ HRAS^G12V^* and *Fh1^Δ/Δ^ HRAS^G12V^*cells, respectively. Fh1-deficient cells and Fh1 WT cells were prepared for injection by resuspension in culture medium and Matrigel (Lapidot, catalog FAL356231) at a 1:1 ratio in a concentration of 0.5 × 10^6^ cells per 20 μL. NSG female mice, 8–10 weeks of age, were injected orthotopically into the kidney capsule with 20 μL cell suspension. An aliquot of blood was collected once from the tail vein before xenografting with tumor cells and then weekly thereafter. Once the tumors could be palpated, tumor growth was monitored once a week by palpating the kidney’s shape, size, and stiffness. When the tumors were superficially visible and weight loss had reached 15% of the animal’s original body weight, the mice were sacrificed by cervical dislocation. The tumors were then removed and preserved for further analysis.

Six- to 8-week-old NSG female mice were used for the PDX experiments. Tumor tissue was obtained from FH-MT patients diagnosed with a germline alteration of FH c.823G>A (p.G275R), whose renal mass was greater than 7 cm. The FH-WT tumor was from ccRCC patient whose right renal mass was greater than 10 cm. Tumor tissues were collected and transferred to a sterile p100 dish containing approximately 10 mL sterile PBS with 1% (vol/vol) penicillin-streptomycin solution. The tissues were cut into fragments of approximately 1.5 mm in diameter for subcutaneous PDX implantation. Each mouse (*n* = 5) was implanted with a PDX of 1 of the 2 genotypes (FH-WT RCC or FH-MT RCC). The fur was shaved off the back of the mouse before PDX implantation. The mice were anesthetized using isoflurane vaporizer by placing them in the induction chamber. Once the mouse was fully anesthetized, it was placed on a tilted surgical board on top of a heating pad. The isoflurane cone was placed on the mouse’s snout. During surgery, the skin was lifted back with a curved forceps, and a 1 cm incision was made by cutting along the skin of the back flank with scissors. Tumor fragments were slowly advanced up to 1.5 cm from the incision. When grafting was completed, the body wall was gently sutured with an absorbable suture. An aliquot of blood was collected from the cheek once before grafting and then weekly thereafter. Subcutaneous tumor growth was monitored every 3 days. When the tumor reached 1–1.5 cm in diameter, the mice underwent an operation to remove the tumor. The tumor were preserved for subsequent IHC and H&E analysis. Aliquots of blood were collected from the cheek on day 1 and day 7 after surgery. Then, the mice were sacrificed by cervical dislocation.

### CT and MRI studies

All CT studies in patients were performed with helical scanners using the triphasic renal protocol for CT systems (VCT LightSpeed, Discovery CT, or Optima 670, GE Healthcare). All patients were administered 400–500 mL water orally 20 minutes before the examination. After unenhanced CT scans, corticomedullary and nephrographic phase images of the diaphragm to the symphysis pubis were acquired, beginning 30 seconds and 65–75 seconds after injection of the nonionic contrast medium (Iopamiro, Bracco Imaging, or Iopromide, Bayer Schering Pharma) at a dose of 2 mL/kg. The imaging parameters for each phase were 120 kVp, a 110–380 mA tube current, and a 5.0 mm slice thickness (1.25 mm with post-processing reconstruction).

Patients were instructed to fast for 4–5 hours prior to MRI scan. Patients were in the supine position for all scans using a 3T magnetic resonance scanner (Ingenia, Philips Medical Systems). The following imaging sequences were used: axial turbo spin-echo T1-weighted imaging, axial and coronal turbo spin-echo T2-weighted imaging, axial diffusion-weighted imaging (DWI), and dynamic contrast-enhanced (DCE) imaging. DWIs with fat suppression were collected using the following single-shot spin-echo echo-planar imaging sequence: repetition time/echo time, 2,000/86 ms; matrix, 152 × 137; in-plane resolution, 2 × 2 mm; section thickness, 5 mm; parallel acquisition with acceleration factor 2; and b values, 0 and 1,000 s/mm^2^. Sensitizing diffusion gradients were applied in the 3 orthogonal directions, and trace DWIs were generated. DCE imaging was performed before and at 3 consecutive points after injection of gadolinium chelate (gadopentetate dimeglumine, Magnevist; Bayer Healthcare) at a dose of 0.1 mmol/kg at a rate of 2 mL/s.

### Statistics

OS rate curves were generated using the Kaplan-Meier method and the log-rank test. Correlation analysis was conducted using Spearman’s correlation. R packages (version 4.0.3) was used to generate the PCA plots, heatmaps, and Venn diagrams. *P* values and the FDR were calculated using a 2-tailed Student’s *t* test or a 2-tailed Wilcoxon test, and multiplicity was adjusted by Bonferroni correction. A *P* value of less than 0.05 was considered statistically significant. Statistical analysis of the quantified plasma markers’ concentration was performed using Python (version 3.9.7). GraphPad Prism, version 8.0 (GraphPad Software), was used to generate graphs and perform statistical analysis unless otherwise indicated.

### Study approval

Animal work was carried out according to protocols approved by the PCRA of Technion, Israel Institute of Technology. Human blood samples were collected from participants from each center. Written informed consent was obtained from each study participant. The study was approved by the ethics committee at RENJI Hospital, Shanghai Jiaotong University and abided by the ethics guidelines of the Declaration of Helsinki.

### Data and code availability

All data and materials supporting the findings of this study are described here and in the supplemental information. The genomic DNA sequencing data were deposited in the National Genomics Data Center (NGDC) Genome Sequence Archive (GSA) database (https://ngdc.cncb.ac.cn/gsa-human/s/921qbMSf) under accession numbers HRA001776 and HRA001736. The metabolomics and lipidomics data were deposited in MetaboLights (https://www.ebi.ac.uk/metabolights/) under accession number MTBLS3628. The detailed methodology for DNA sequencing, metabolomics, and lipidomics is provided in the Supplemental Methods.

## Author contributions

LZ, EG, and JZ designed and supervised the study, reviewed the data, and wrote the manuscript. LZ, JFG, and WTZ interpreted the large-scale metabolomics data and performed the statistical analysis. LZ and ZRZ performed the cell experiments and protein work. YZX, ZYW, JZ, GYW, YZX, LZ, and EG reviewed, interpreted, and verified the clinical information and data. ZYW and WH performed pathological and IHC analyses. GYW conducted the radiological analyses. YZX, LZ, HGQ, and HW contributed to the clinical study and obtaining patients’ samples. JFG, WTZ, and LZ conducted the bioinformatics and statistical analyses. XYW, IA, and LZ performed the metabolomics work. TS, IA, and EG performed the mouse model work. All authors have approved the final version of the manuscript.

## Supplementary Material

Supplemental data

Supplemental table 1

Supplemental table 2

Supplemental table 3

Supplemental table 4

## Version 1 04/13/2023

In-Press Preview

## Version 2 06/01/2023

Electronic publication
