# Supplementary material for: Circulating succinate-modifying metabolites accurately classify and reflect the status of fumarate hydratase–deficient renal cell carcinoma
Source: J Clin Invest. 2023 Jun 1;133(11):e165028. doi: 10.1172/JCI165028 (PMC10232000; doi:10.1172/JCI165028)
Supplement: Supplemental data [file jci-133-165028-s193.pdf]

Supplemental Information

Supplemental Methods

IHC

IHC was created by archived, formalin-fixed, paraffin-embedded tumor sections from a total of 811 renal neoplasms for FH-WT RCC, which were obtained from three hospitals across China. They were analyzed by Hematoxylin and Eosin (H&E) and Immunohistochemistry (IHC) staining for FH and 2SC checking FH status.

IHC was carried out using the EnVision kit (Dako) according to the manufacturer's protocol. The following antibodies and respective specifications were used: primary anti-FH (Proteintech, Cat# 11375-1-AP, 1:200) and an anti-2SC (Discoveryantibodies, Cat# crb2005017, 1:1000). Staining for 2SC were scored as - if negative; + if focal (<50% of cells reactive) or diffuse (>50% of cells reactive) but of weaker intensity; or ++ if diffuse positive (>50% of cells reactive) with strong intensities. Negative staining in the adjacent normal renal parenchyma was considered an internal negative control. A positive IHC result was considered when both antibodies showed a pattern indicating FH deficiency [2SC(++); FH(-)]; a negative IHC result was when FH antibody showed retained FH expression and 2SC was not expressed [2SC(−); FH(+)]. An undetermined intermediate IHC result was considered when at least only one antibody showed FH deficiency, whereas the other was equivocal or negative [2SC (++); FH(+)], or [2SC(+); FH(+)], or [2SC(+); FH(−)], or [2SC(-);FH(−)]. All cases which demonstrated FH deficiency [2SC(++); FH(-)] or undetermined intermediate results underwent further genetic analysis of FH status. DNA was extracted from patients' peripheral blood leukocytes or macro-dissected FFPE tissue. Genetic testing was performed using Whole Exome Sequencing (WES) for small nucleotide variants, and multiplex ligation-dependent probe amplification (MLPA) for detection of large-scale deletions.

Whole Exome Sequencing

<u>DNA Library Preparation:</u> For exome sequencing, we fragmented 1–3 μg of genomic DNA, extracted from each sample, to an average size of 180 bp with a Bioruptor sonicator (Diagenode). Paired-end sequencing libraries then were prepared using a DNA sample prep reagent set 1 (NEBNext). Library preparation included end repair, adapter ligation and PCR enrichment, and was carried out as recommended by Illumina protocols.

<u>Targeted genes enrichment and sequencing:</u> The amplified DNA was captured use GenCap WES capture kit (MyGenostics GenCap Enrichment technologies). The DNA probes were designed to tile along the exon regions of the 23000~ genes. The capture experiment was conducted according to manufacturer's protocol. In brief, 1 μg DNA library was mixed with Buffer BL and GenCap WES probe (MyGenostics, Beijing, China), heated at 95°C for 7 min and 65°C for 2 minutes on a PCR machine; 23 μL of the 65°C pre warmed Buffer HY (MyGenostics Inc, Beijing, China) was then added to the mix, and the mixture was held at 65°C with PCR lid heat on for 22 hours for hybridization. 50 μL MyOne beads (Life Technology) were washed in 500 μL 1×binding buffer for 3 times and resuspended in 80 μL of 1× binding

buffer. 64 μL 2× binding buffer was added to the hybrid mix, and transferred to the tube with 80 μL of MyOne beads. The mix was rotated for 1 hour on a rotator. The beads were then washed with WB1 buffer at room temperature for 15 minutes once and WB3 buffer at 65°C for 15 minutes three times. The bound DNA was then eluted with Buffer Elute. The eluted DNA was finally amplified for 15 cycles using the following program: 98°C for 30 s (1 cycle); 98°C for 25 s, 65°C for 30 s, 72°C for 30 s (15 cycles); 72°C for 5 minutes (1 cycle). The PCR product was purified using SPRI beads (Beckman Coulter) according to manufacturer's protocol. The enrichment libraries were sequenced on Illumina HiSeq X ten sequencer for paired read 150bp.

Bioinformatics analysis: After sequencing, the raw data were saved as in FASTQ format, then followed by the bioinformatics analysis: First, Illumina sequencing adapters and low quality reads (<80bps) were filtered by cut adapt. After quality control, the clean reads were mapped to the UCSC hg19 human reference genome using BWA. Duplicated reads were removed using pi card tools and mapping reads were used for variation detection. Second, the variants of SNP and In Del were detected by GATK Haplotype Caller, then using GATK Variant Filtration to filter variant, the filtered standard as follows: a) variants with mapping qualities < 30; b) the Total Mapping Quality Zero Reads < 4; c) approximate read depth < 5; d) QUAL<50.0; e) phred-scaled p-value using Fisher's exact test to detect strand bias > 10.0. After above two steps, the data would be transformed to VCF format, variants were further annotated by ANNOVAR and associated with multiple databases, such as: 1000 genome, ESP6500, dbSNP, EXAC, Inhouse (MyGenostics), HGMD, and predicted by SIFT, PolyPhen-2, MutationTaster, GERP++.

Pathogenic Variants Selection: In this course, five steps were used to select the potential pathogenic mutations: (i) Mutation reads should be more than 5, mutation ratio should be no less than 30%; (ii) Removing the mutation, the frequency of which showed more than 5% in 1000 g, ESP6500 and Inhouse database; (iii) If the mutations existed in Normal database (MyGenostics), then dropped; (iv) Removing the synonymous mutation. (v) After (i), (ii), (iii), if the mutations were synonymous and they were reported in HGMD, which kept them. Following the above steps, the mutations which were kept should be the pathogenic mutations.

Expanded Validation: Filtered candidate variants were confirmed by Sanger sequencing. The coding exons that contain the detected mutations were amplified with Ex Taq DNA polymerase (Takara, Dalian). Purified PCR samples were sequenced on an ABI 3730 Genetic Analyzer (Applied Biosystems, CA). Sequence traces were analyzed using the Mutation Surveyor (Softgenetics, PA). The mutations of family members were confirmed by the same procedure.

The *FH* gene mutation database analyses

The published FH mutations' information were obtained from three resources: 1, The Human Gene Mutation Database; 2, https://databases.lovd.nl/shared/genes/FH; 3, Clinvar from NCBI (https://www.ncbi.nlm.nih.gov/clinvar/). After archiving all data files, the related references were manually curated. Information of chromosome loci, amino acids' locations and occurrences of variants were documented.

Plasmid construction and stable expression in cell lines

Full length wildtype and mutated *FH* human cDNA were cloned into GV341 vector digested with Age I/Nhe I. Plasmid GV341, pAX2 and pMD2 were co-transfected into HEK293T cells using Lipofectamine 2000 (Life Technology, Cat# 1668019) to produce viruses. Viruses infected with *Fh1*$^{-/-}$ cells for 24h and transformed cells were then selected in 1 μg/mL of puromycin for 5-7 days. Protein expression of FH was confirmed by immunoblotting with Flag and FH antibodies, the mRNA levels were detected by qPCR. The Materials and Reagents details were listed in Supplementary Table S4.

Genomic DNA, RNA isolation and qPCR analysis

Cells were harvested and washed with PBS ×3 times. Genomic DNA was isolated using DNA Mini Kit (QIAGEN, Cat# 80284) and RNA was extracted by using TRIzol™ (Invitrogen, Cat# 15596026). Genomic DNA was gathered to determine the different genotypes of the different cell lines using the related primers. The PCR was performed using 2×EasyTaq PCR SuperMix (TransGenBiotech, Cat# AS111-01), and then products were separated in a 1% agarose gel stained with a nucleic acid gel stain.

For real-time qPCR analysis, 2μg of total mRNA was retro-transcribed into cDNA using a PrimeScript™ II 1st Strand cDNA Synthesis Kit (Takara, Cat# 6110A), and using a Cham QTM Universal SYBR qPCR Master Mix (Vazyme, Cat# Q511-02) to make up the qPCR reaction. The Reaction system was 0.2μM primers mixed with ROX dye and 5μL of 1:10 dilution of cDNA in a final volume of 20μL. Real-time PCR was performed on the CFX ConnectTM Real-Time PCR System (BIO-RAD). The relative quantification of each mRNA was carried out with the comparative threshold cycle method using GAPDH for housing control.

Immunoblotting

RIPA buffer (150mM sodium chloride, 1.0% Triton X-100, 1% sodium deoxy cholate, 0.1% SDS and 50mM Tris, pH 7.4) supplemented with protease and phosphatase inhibitor cocktail (Sigma, Cat# PPC1010) was used to lyse 1×10^6 cells. The concentration of protein was detected with the Bicinchoninic Acid Assay (ThermoFisher, Cat# 23225). Proteins were loaded into 10% SDS-PAGE gels in equal amounts and separated by electrophoresis ally. After that, proteins in the gels were blotted onto 0.45 mm nitrocellulose (Millipore) and probed with relevant antibodies, which were diluted in 3% BSA: FLAG M2 (Sigma, Cat# F3165, RRID:AB_259529), FH (Cell Signaling Technology, Cat# 4567, RRID:AB_11178522), GAPDH (Cell Signaling Technology , Cat# 5174, RRID:AB_10622025), β-Actin (Cell Signaling Technology, Cat# 8457, RRID:AB_10950489), ADSL (Abcam, Cat# ab154182), DPEP1 (Invitrogen, Cat# PA5-52984), GGT1 (Abcam, Cat# ab175384), GGT5 (Sino Biological, Cat# 16136-T56), DPEP2 (Sino Biological, Cat# 13806-RP01), ANPEP (Sino Biological, Cat#10051-R122) and LAP3 (Sino Biological, Cat# 15480-T62). The secondary HRP-conjugated antibodies (Cell Signaling Technology, Cat# anti-mouse 7076, RRID: AB_330924 and anti-rabbit 7074 at 1:5000 dilution in 3% BSA both, RRID: AB_2099233) were used to recognize the primary antibodies respectively. ECL (GE Healthcare, Cat# RPN 2209) was used for chemi-luminescence detection.

Fumarate hydratase Activity Assay

This assay was carried on fumarate hydratase activity assay kit (Biovision, Cat# K596). Cells 1.5×10^6 were used and homogenized with 100 μL ice-cold fumarate hydratase assay buffer. The following steps were referred to the assay protocols.

In-house synthesis of internal standards of $^{13}C_3{}^{15}N$-suc-cys and $^{13}C_2{}^{15}N$-suc-ado

$^{13}C_3{}^{15}N$-suc-cys was obtained by reacting $^{13}C_3{}^{15}N$-cysteine (Cambridge Isotope Laboratories, Cat# CNLM-3871) with fumaric acid (Sigma, Cat# 47910) in a 30% NaOH aqueous solution at room temperature overnight. The purification was achieved by applying a C18 preparative column. The purity was checked by HPLC, MS and NMR.

$^{13}C_2{}^{15}N$-suc-ado was synthesized in four steps. In the first step, the adenine nucleotide of compound 1 after the esterification reaction was reacted with oxalyl chloride (Sigma, Cat# 221015) under the methylene chloride (Sigma, Cat# PHR1557) at 0 to 40 °C for 3 hours to obtain compound 2; In the second step, the product of the first step was reacted with 1,4-$^{13}C_2$,^{15}N-asparagine (Cambridge Isotope Laboratories, Cat# CNLM-7818) in potassium carbonate (Sigma, Cat# 367877) dissolved in N, N-dimethylformamide (DMF, Sigma, Cat# 227056) at a reaction temperature of 120 °C for 8 hours to obtain compound 3; Compound 4 was obtained by removing the acetyl group in 10% sodium hydroxide aqueous solution: methanol = 1: 5 at 40 °C for 5 hours; In the fourth step, Pd/C was used as a catalyst for the addition of hydrogen, and the reaction was performed overnight in methanol: water = 1:1 (v/v), and finally $^{13}C_2{}^{15}N$-suc-ado was obtained. The purification was achieved by using a C-18 preparative column. The purity was checked by HPLC, MS and NMR.

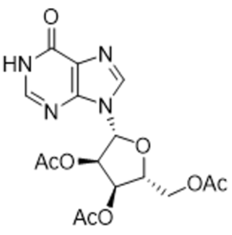

compound 1

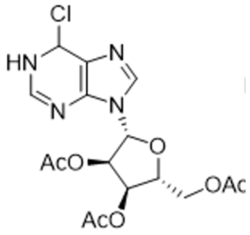

compound 2

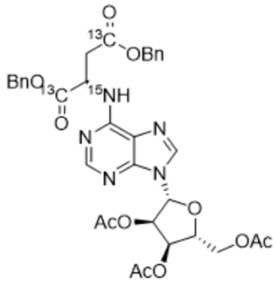

compound 3

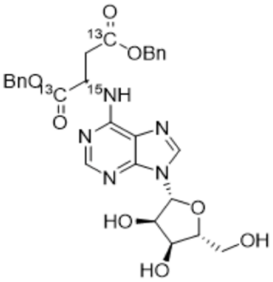

compound 4

Metabolic extraction of cell lines or blood

Cells (5×10^5) were plated onto six-well plates and cultured in standard medium for 24 h. For the intracellular metabolic analysis, cells were quickly washed for three times with PBS to remove contaminations from the media. The PBS was thoroughly aspirated and cells were lysed by adding a precooled extraction solution (80% methanol, ES). The cell number was counted in a parallel control dish, and cells were lysed in 1 ml of ES per 1×10^6 cells. The cell lysates were vortexed for 5 min at 4 °C and immediately centrifuged at 16,000g for 15 min at 0 °C. The supernatants were collected and analyzed by LC-MS. For Isotope Tracing Experiments Cells (5×10^5) were plated onto six-well plates and cultured in standard medium for 12 h. The medium was then replaced by fresh medium supplemented with 2 mM U-^{13}C-Glutamine or for

the 24 h. The mice were injected via the tail vein with indicated amounts of exogenous metabolites. Blood samples were taken intraorbitally at the indicated time after the injection.

Metabolomics analysis (untargeted)

Plasma sample preparation: Plasma was prepared from fresh blood treated with anti-coagulate EDTA, aliquoted, and stored at -80ºC. Within discovery study, 30 plasma samples from 30 separate individuals were performed with untargeted metabolomics, which included FH-deficient (n=10), FH-wildtype (n=10) and normal control (n=10). Plasma (50 µL) was mixed with 950 µL of an acetonitrile: water (4:1, v/v) solvent, vortexed for 15 min at 4ºC. The supernatant was collected after centrifugation at 15,000 rpm for 15 min at 4ºC. A quality control (QC) sample was generated by pooling all the plasma samples from all samples.

MS acquisition and Chromatography: A thermo Q-Exactive plus mass spectrometer (Thermo Scientific, USA) was operated in full MS-scan mode (acquisition from m/z 70 to1, 000) with a scan resolution set at 35,000. The MS/MS spectra of the QC samples were acquired under different fragmentation energies (30 NCE, 40 NCE and 50 NCE) for the top 20 parental ions. The separation was performed using ZIC-pHILIC column (150 mm × 2.1 mm, 5µm, Merck) and ZIC-HILIC column (150 mm × 2.1 mm, 5µm, Merck). For the ZIC-pHILIC column, the mobile phases are 20mM ammonium carbonate (Sigma Aldrich Cat# 379999) and 0.2% ammonia (Fluka Cat# 60-003-11) in water (phase A) and acetonitrile (Fisher Chemical, Cat# A998-1, phase B). Metabolites were eluted from the column at a flow rate of 0.25 mL/min. 0-1.5 min with 90% B, 1.5-25 min for 90% to 40% B, 25-28 min for 40% to 90% B, 28-33 min 90% B. The oven temperature was set to 30ºC. Mobile phases for the ZIC-HILIC consisted of A (0.1% formic acid in water) and B (0.1% formic acid in acetonitrile). The flow rate was 0.2 mL/min. The elution gradient was as follows: 0-2 min with 80% B, 2-21 min for 80% to 20% B, 21-25 min 20% B, 25.1min for 20% to 80% B, and 25.1-31 min 80% B. The injection volume for both columns was 3.0 µL.

Metabolomics Data Processing: Metabolomic features were extracted with compound discoverer 3.2 (Thermo Fisher Scientific, Waltham, USA). The peak deconvolution was performed on the default settings with our own modifications. We set the peak intensity threshold to 10000 in order to eliminate background peaks. Data-dependent ($ddMS^2$-top20) MS/MS data were obtained on selected samples or pooled samples for identification purposes. Metabolite identification was performed using a two-step approach. First, we used self-built standard reference library, which contains accurate mass (m/z ± 5 ppm), retention time (RT ± 1.0 min) and MS/MS spectral patterns. Second, further remaining metabolites were identified based on accurate mass, isotope pattern and MS/MS spectra against public databases, including HMDB, KEGG, mzCloud and MassBank. In practice, the mass of metabolites was matched against the public database: HMDB and KEGG, using the cutoff value of 5ppm. Next, for MS/MS data, if one metabolite feature matched multiple MS/MS spectra, then all matched MS/MS spectra were used for the identification. In the rare cases, when a given metabolic feature was matched differently between different matching methods, we chose the matching based on the identification level: Standards (accurate mass + retention time+ MS/MS > MS/MS (accurate mass + MS/MS) > HMDB/KEGG (accurate mass). Peak shapes in compound

discoverer 3.2 were individually evaluated. If the peak shapes appeared unreliable (e.g. asymmetric), thermo LCQUAN software was used to integrate the peak area.

Regularized Partial Correlation Network: A regularized partial correlation network was constructed by R-igraph. We chose the following metabolites from two categories: first, top 15 metabolites (FDR value <0.05) from FH-deficient RCC versus normal control; second, metabolites (FDR <0.05) from FH-deficient RCCs versus FH-wildtype RCC. Each node represents a metabolite, and each edge represents the strength of partial correlation between two nodes. Each metabolite was mapped onto established biochemical groups. The edge thickness represents the partial correlation coefficients. The size of each node represents correlations to the tumor mass.

Lipidomic analysis (untargeted)

Plasma sample preparation: The 30 samples from same plasma IDs that were used in the discovery study were used for lipidomic studies. An aliquot of of plasma (100 µL) was added to 200 µL of water, then mixed with 240 µL of pre-chilled methanol and vortexed for 5 min, and the mixed with 800 µL of Methyl tert-butyl ether (MTBE) and vortexed for 5 min. Left at room temperature for 30 min, and then centrifuged at 14000 g at 10°C for 15 min. The upper organic was removed and dried with nitrogen, and 200 uL isopropanol was added and the sample was vortexed The sample was then centrifuged at 14000g at 10°C for 15 min, and the supernatant removed for analysis by LCMS. A QC sample was generated by pooling aliquots from all the plasma samples.

MS acquisition and Chromatographic conditions: A thermo Q-Exactive plus mass spectrometer (Thermo Scientific, USA) was operated in full scan mode (acquisition from m/z 200 to1, 800 for positive and negative mode, respectively) with a scan resolution set at 70,000 at m/z 200. The MS/MS spectra of the each sample were acquired under various fragmentation energies (20 NCE, 25 NCE and 30 NCE) for the *ddMS*^{2}-top10. An ACQUITY CSH C18 (2.1 mm × 100 mm, 1.7 µm, Waters) column was used on a Nexera LC-30A ultra-high performance liquid chromatography system. The column temperature was 45°C; the flow rate 300 µL/min; the injection volume 2 µL. Mobile phase composition A: 10 mM ammonium formate and acetonitrile aqueous solution (acetonitrile: water=6:4, v/v), B: 10 mM ammonium formate and acetonitrile isopropanol solution (acetonitrile: isopropanol=1:9, v/v). The gradient elution procedure was as follows: 0-7 min, 30% B; 7-25 min, B changes linearly from 30% to 100%; 25.1-30 minutes, 30% B. The samples were placed in the autosampler at 10°C during the analysis.

Lipidomic Data Processing: Lipid species were identified using the LipidSearch v4.2 (Thermo Fisher Scientific, Waltham, USA) to process the raw data. LipidSearch database contains more than 30 lipid classes, with more than 1,700,000 fragments. Adducts of +H, +NH_4 were selected for positive mode searches, while -H, -CH_3COO were selected for negative mode searches since ammonium acetate was used in the mobile phases. The identification of lipids mainly carried out four steps. First, the parental ions and fragment ions were matched with the LipidSearch (mass tolerance = ±5 ppm). Second, the matched peaks were grouped based on the same molecular formula

and retention time. Third, grades A, B, C, D were used to score each grouped peak (A=10, B=5, C=1, D=0.5; No ID = 0). Grade A indicates the full information including the head group, the carbon number of fatty acid chains, the degree of unsaturation and the position of the double bond. Grade B indicates the partial information including the head group, the carbon number of the fatty acid chains and the degree of unsaturation. Grades C and D indicates the limited information including the head group of the lipid and the carbon number of the chain. Finally, the scores from highest to lowest were sorted, and groups with lower scores will be eliminated.

LC-MS quantification of plasma marker metabolites

$^{13}C/^{15}N$ labeled suc-cys and suc-ado were used as internal standards (in-house synthesis) to reduce the effect of ion suppression. The internal standard working solution was prepared by diluting the stock solution with acetonitrile: water (80:20 v/v). 800 µL of $^{13}C_3{}^{15}N$-suc-cys (10 µg/mL) and 650 µL of $^{13}C_3{}^{15}N$-suc-ado (10 µg/mL) were added to 500 mL solution (acetonitrile: water = 4:1 v/v) to obtain stable labeled internal standard (SIL) solution (containing 16 ng/mL of $^{13}C_3{}^{15}N$-suc-cys and 13 ng/mL of $^{13}C_3{}^{15}N$-suc-ado). The working solution of the calibration curve was prepared by diluting 1mg/mL ^{12}C-suc-cys and ^{12}C-suc-ado standard stock solutions with SIL solution. First add the diluted standard solution (25 µL) and blank plasma (25 µL) without suc-cys and suc-ado to 450 µL of extraction buffer. The mixed solution was vortexed at room temperature for 10min, and then centrifuged at 20000 g for 20 min. Thus, ^{12}C-suc-cys and ^{12}C-suc-ado working solutions with different concentration levels were obtained by taking the supernatant after centrifugation. The linear range of ^{12}C-suc-cys was 0.5~500 ng/mL, and the linear range of ^{12}C-suc-ado was 0.1~200 ng/mL. The standard curve was represented with ^{12}C-concentration as the abscissa and $^{12}C/^{13}C$-ratio as the ordinate.

Aliquots of 50 µL of human plasma were extracted with 950 µL SIL solution (containing 16 ng/mL of $^{13}C_3{}^{15}N$-suc-cys and 13 ng/mL of $^{13}C_3{}^{15}N$-suc-ado). The tube was vortex mixed for 10 min. After centrifugation at 20000 g for 20 min, 900 µL of the supernatant were transferred to a 1.5 mL new tube and centrifugation was continued for 5min to obtain a plasma extract containing the ^{13}C and ^{15}N labelled internal standards. The resulting solution was used for LC-MS analysis. Sample were analyzed using a Q-Exactive plus mass spectrometer (Thermo Fisher Scientific, USA) linked to Ultimate 3000 UPLC (Dionex, Sunnyvale, USA). A ZIC-HILIC column (150 mm × 2.1 mm, 5µm, Merck) was utilized on this application. The elution conditions were as described above.

Construction of the recombinant human proteins and in vitro assay of activities

All recombinant human proteins were firstly searched online to ascertain commercial availability. After purchasing the available ones, their activities were tested in vitro on our own platform. If no activities were found, desired proteins were constructed in house. Finally, by using the expressing system, like HEK293 or baculovirus, we constructed 7 enzymes, including glutamylcyclotransferase1 (GGT1), glutamylcyclotransferase5 (GGT5), glutathione specific gamma-glutamylcyclotransferase1 (CHAC1), DPEP1, DPEP2, ANPEP and LAP3. Of them, human GGT1, GGT5, DPEP1, DPEP2 and ANPEP with N/C His tag were all overexpressed in HEK293 cells. The sequence of amino acids and cDNA details has been provided (Supplementary Table S4). All constructs were generated by standard PCR-ligation techniques

and the constructed vectors were validated by sequencing. The correct plasmids were amplified and transferred to cells. For transient transfection, the plasmids were mixed with Hieff Trans^{TM} Liposomal Transfection Reagent at an optimal ratio and then added into the flask containing HEK293. The cells were cultured in a serum-free medium and maintained in Erlenmeyer flasks on an orbital shaker by a suitable stirring speed at 37ºC for 6 days. For protein purification, cells were collected and the supernatant was loaded onto an affinity purification column. The protein concentrations of the final products were determined by UV or BCA assays. The purities of the final products were analyzed by SDS-PAGE. The human proteins CHAC1 with N-His tag and LAP3 with C-His tag were expressed using baculovirus expression vector system (BEVS). All target cDNAs were inserted into vector followed by the generation of the recombinant baculovirus. Recombinant vectors were amplified into cells to prepare high titer virus stocks. For protein expression, cells were infected with recombinant baculovirus and target protein was expressed under optimal conditions. Cell pellets were collected and homogenized. The lysate supernatant was collected and loaded onto an affinity purification column. Target proteins were eluted from the column using optional elution fractions. The protein concentration of the final products were determined by UV or BCA assays. The purities of the final products were analyzed by SDS-PAGE.

In vitro assay of activity was performed separately as follows. 0.5μg of recombinant human GGT1 was mixed with the substrate of suc-GSH (200μM) or GSH (200μM) in 250μL of buffer (50mM Tris, 0.05% Tween 20, pH=8.0). 0.5μg of GGT5 was mixed with the substrate of suc-GSH (200μM) or GSH (200μM) in 250μL of buffer (50mM Tris, 0.05% Tween 20, pH=8.0). 0.025μg of gamma-glutamylcyclotransferase (GGCT) was mixed with the substrate of suc-GSH (500μM) or γ-L-glutamyl-L-alanine (γ-L-glu-ala 500μM) in 250μL of buffer (50mM Tris, pH= 9.0). 0.5μg of CHAC1 was mixed with the substrate of suc-GSH (200μM) or GSH (500μM) in 250μL of buffer (50mM Tris, pH=8.0). 0.5μg of DPEP1 was mixed with suc-cys-gly (100μM) or Leukotriene D4 (LTD4, 20μM) or cys-gly (200μM) in 200μL of buffer (100 mM Tris, pH=8.0). 0.5μg of CNDP2 was mixed with suc-cys-gly (100μM) or L-carnosine (β-ala-his, 500μM) or cys-gly (500μM) in 200μL of buffer (50 mM Tris, 0.1 mM $MnCl_2$, pH=9.0). 0.5μg of ANPEP was mixed with suc-cys-gly (100μM) or ala-AMC (100μM) or cys-gly (500μM) in 200μL of buffer (50 mM Tris, pH=7.0). 1μg of DPEP2 was mixed with suc-cys-gly (100μM) or LTD4 (20μM) in 200μL of buffer (100 mM Tris, pH=8.0). 1μg of LAP3 was mixed with suc-cys-gly (100μM) or L-leu-leu (200μM) in 200μL of buffer (50 mM Tris, 2mM $ZnCl_2$, pH=8.0). In addition, if the substrates or products contained free thiol (–SH), extra 5mM DDT was supplemented. The mixtures were incubated at 37ºC bath and at the time points 0, 5, 10, 30, 60min of the experiment. The reaction was terminated by quenching solution (see quenching method for metabolomics) and the extracts were spun down at maximum speed for 15 min. The supernatant was subsequently analyzed by LC-MS. For substrate blanks, only the indicated assay buffer and substrate were incubated in a mix without enzymes.

Mouse tissue protein extraction

Frozen tissues were cut on glass plate under dry ice and then weighed. An aliquot of RIPA (containing a protease inhibitor cocktail) was added to each tube (stainless steel beads added) to make 1mg of tissue in every 5 μL of RIPA solution. The samples were homogenized using a

tissue lyser with for 60s at 70 Hz by 3 times. The beads were removed and the homogenized tissue was transferred to a new tube. The tube was then centrifuged at 12000 rpm at 4ºC for 15min. Protein in the supernatants was quantified using the BCA assay.

Statistics

Overall Survival rate curves were performed using the Kaplan-Meier method and the log-rank test. Correlation analysis was conducted by Spearman's correlation. R packages (http://www.R-project.org, version 4.0.3) were used for PCA-plot, Heatmap-plot and Venn diagrams. Statistical significance of *P* value or false discovery rate (FDR) was calculated using Student's t test (two-tailed) or Wilcoxon test (two-tailed) and multiplicity was adjusted by Bonferroni Correction. A *P* value less than 0.05 was considered significant. Statistical analysis of the quantified plasma markers' concentration was performed using Python v.3.9.7. GraphPad Prism Software (version 8.0) was used to generate graphs and perform statistical analysis unless otherwise indicated.

Supplemental Figure 1

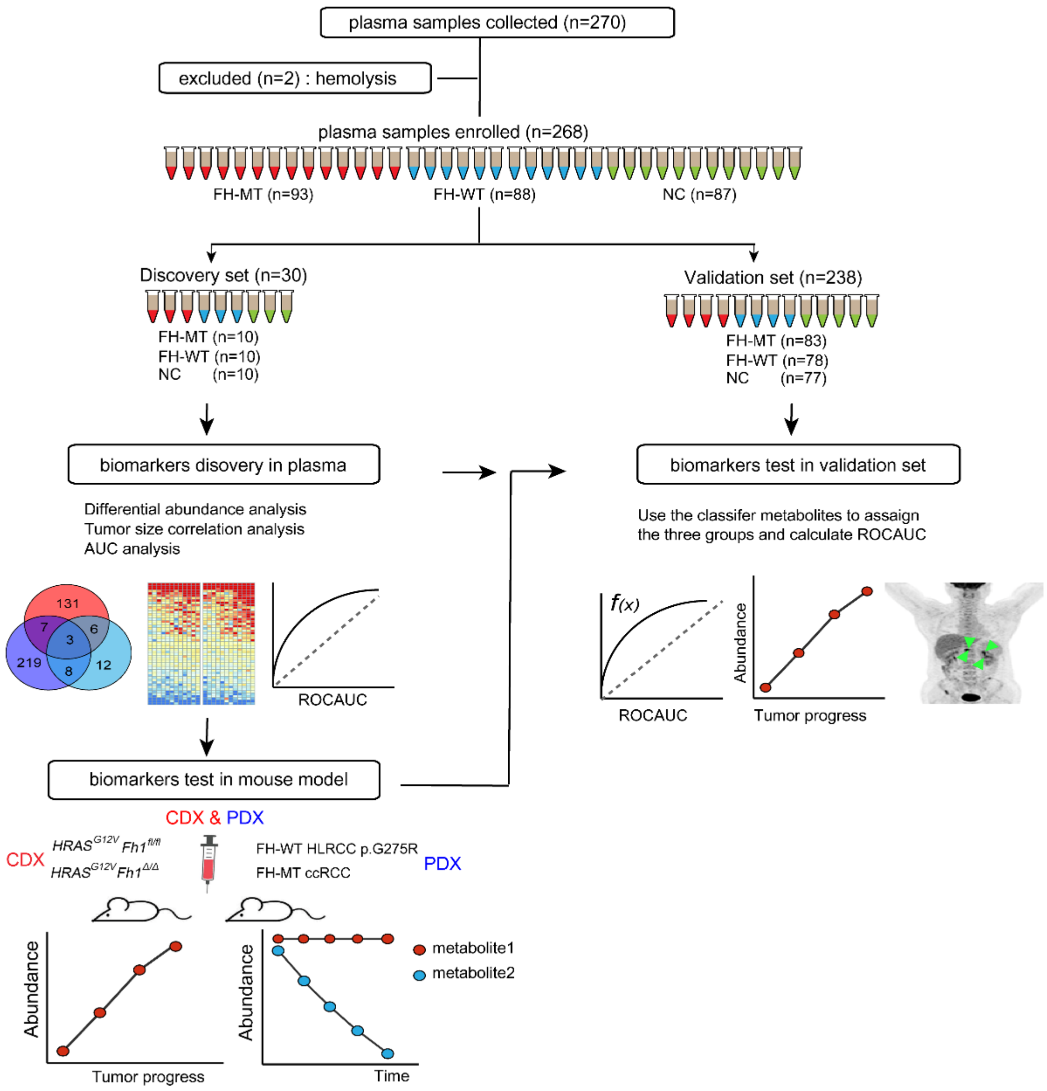

Supplemental Figure 1. Flowchart for plasma biomarkers studies in renal cell carcinoma. A highly sensitive, high-throughout, MS-based metabolomics technology were used to identify liquid metabolic biomarkers in n=268 human plasma samples. The accompanying in vitro biochemical and in vivo mouse model were used to study the production mechanism of plasma biomarkers during the tumor growth.

Supplemental Figure 2

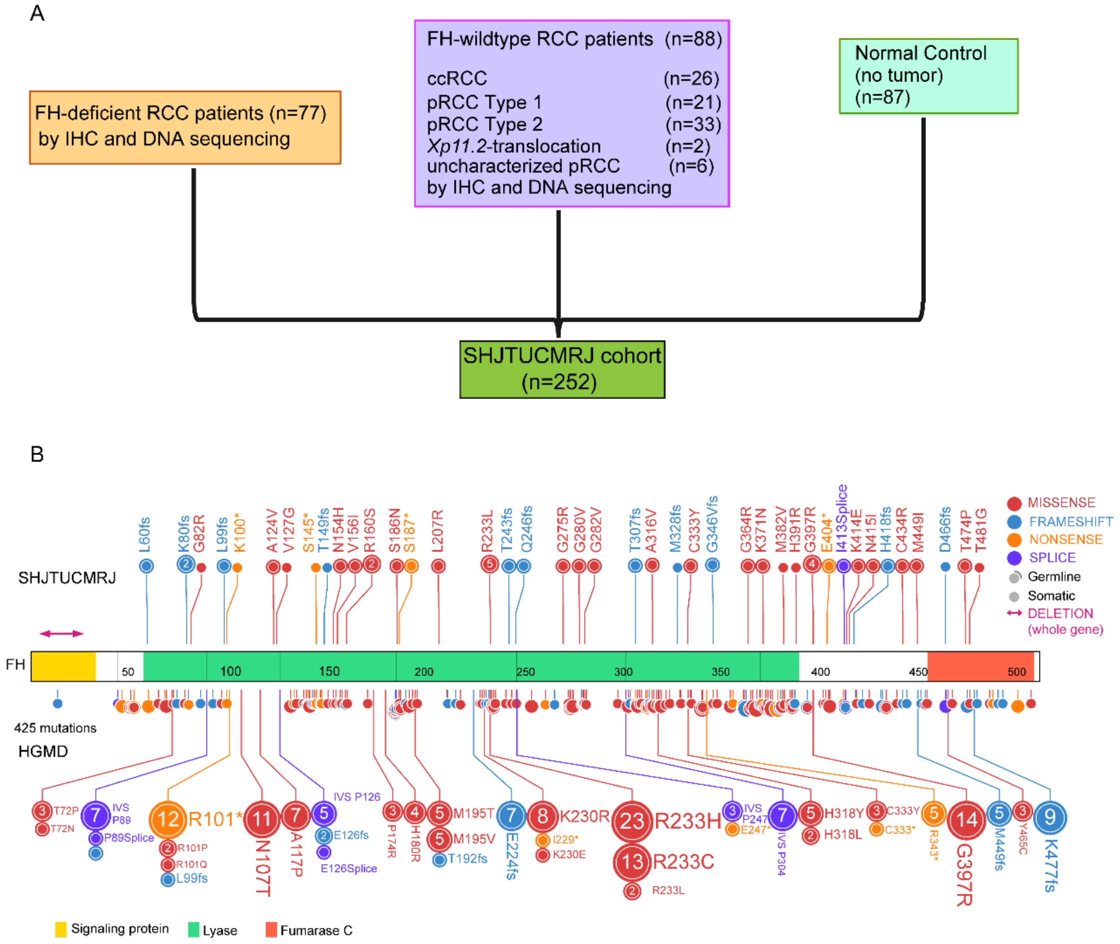

Supplemental Figure 2. The genetic features of various FH mutations in the FH-MT section of the SHJTUCMRJ RCC cohort.

(A) A CONSORT diagram describing the construction of the SHJTUCMRJ cohort. Abbreviations: Clear cell renal carcinoma (ccRCC), papillary renal cell carcinoma (pRCC).

(B) Distribution spectrum of FH mutations (genomic loci) of FH-deficient patients. (Upper panel: 70 FH variants in this SHJTUCMRJ cohort. Lower panel: A summary of 425 FH variants from the Human Gene Mutation Database (HGMD). The encircled numbers indicate the occurrence of specific variants.

Supplemental Figure 3

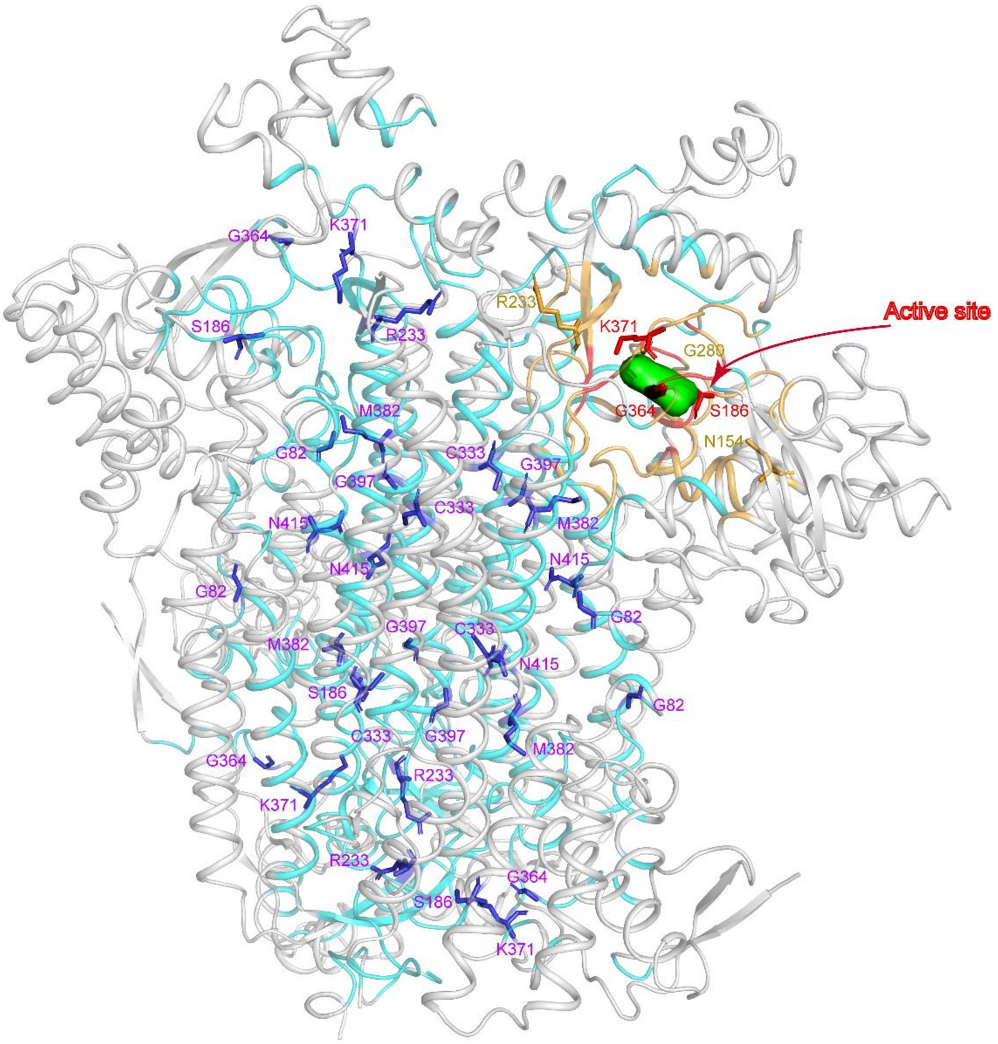

Supplemental Figure 3. Distribution of representative pathogenic missense mutations in the SHJTUCMRJ cohort in the homotetrameric FH holoenzyme. The indicated amino acid substitution are placed on one of three domains: the active site (red), near (10 Å) the active site (yellow), and on the subunit interaction sites (purple-blue).

Supplemental Figure 4

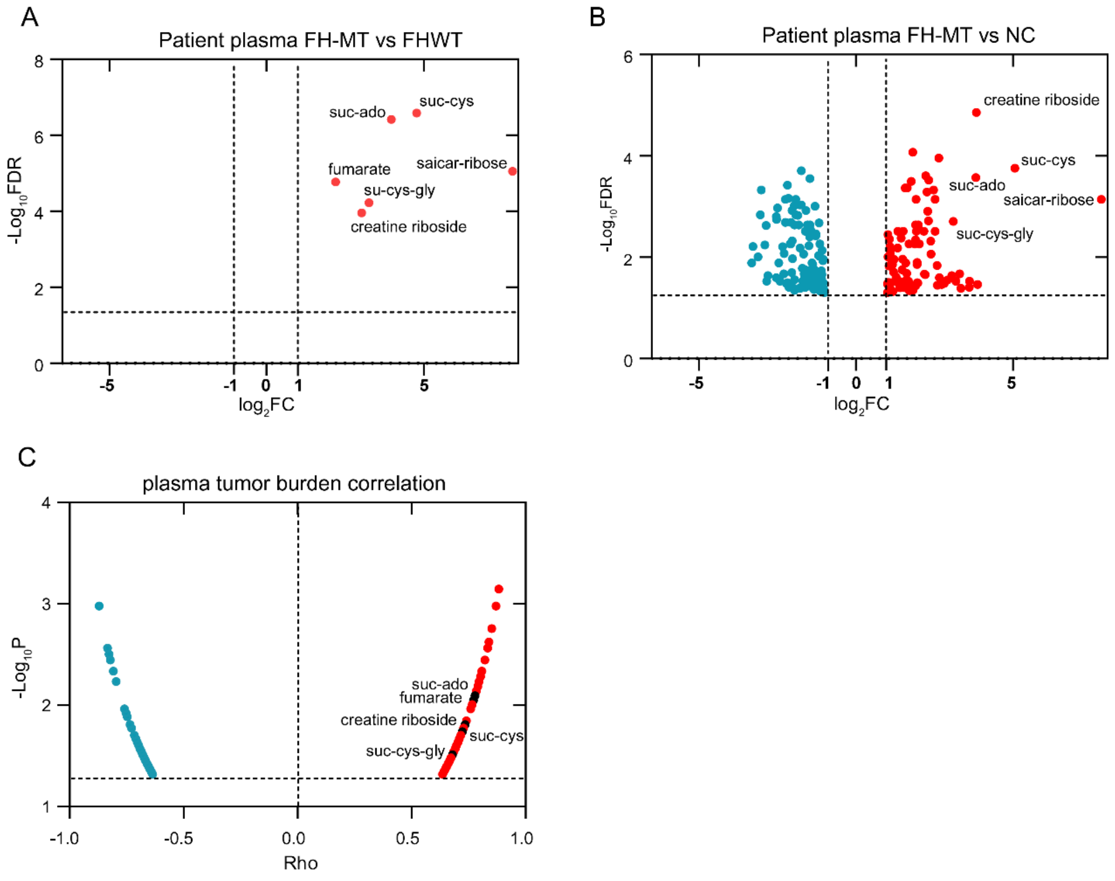

Supplemental Figure 4. Volcano plot analysis of plasma metabolites in the discovery set. (A) FH-MT versus FH-WT **(B)** FH-MT versus NC **(C)** Correlation coefficient analysis between tumor mass and the levels of plasma metabolites.

Supplemental Figure 5

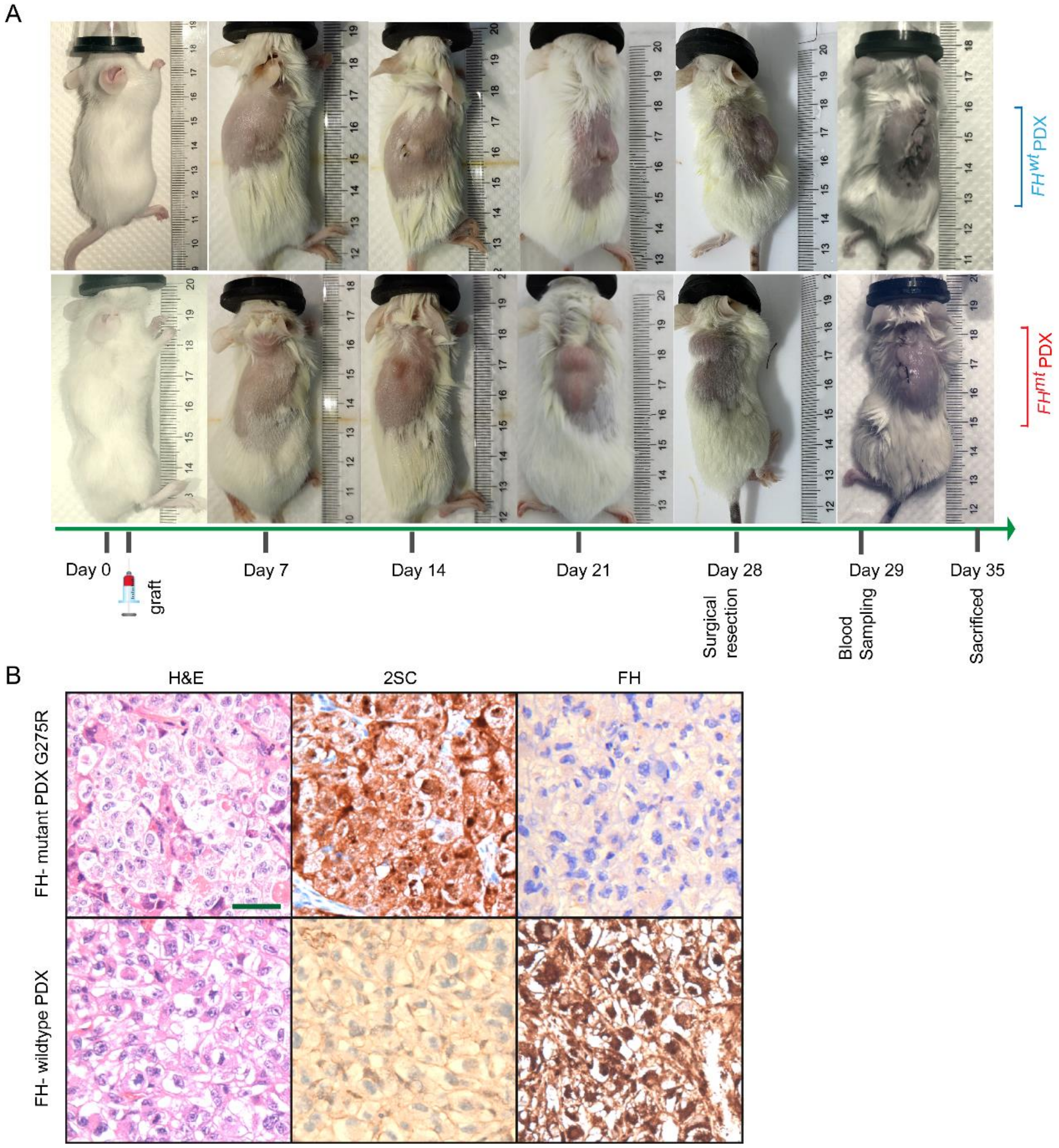

Supplemental Figure 5. The generation of subcutaneous PDX mice.

(A) The representative images of tumor development of mice engrafted with PDX tumors subcutaneously. **(B)** H&E and IHC staining for 2SC and FH on the tissue from mouse PDX tumor. Scale bars: 50μm.

Supplemental Figure 6

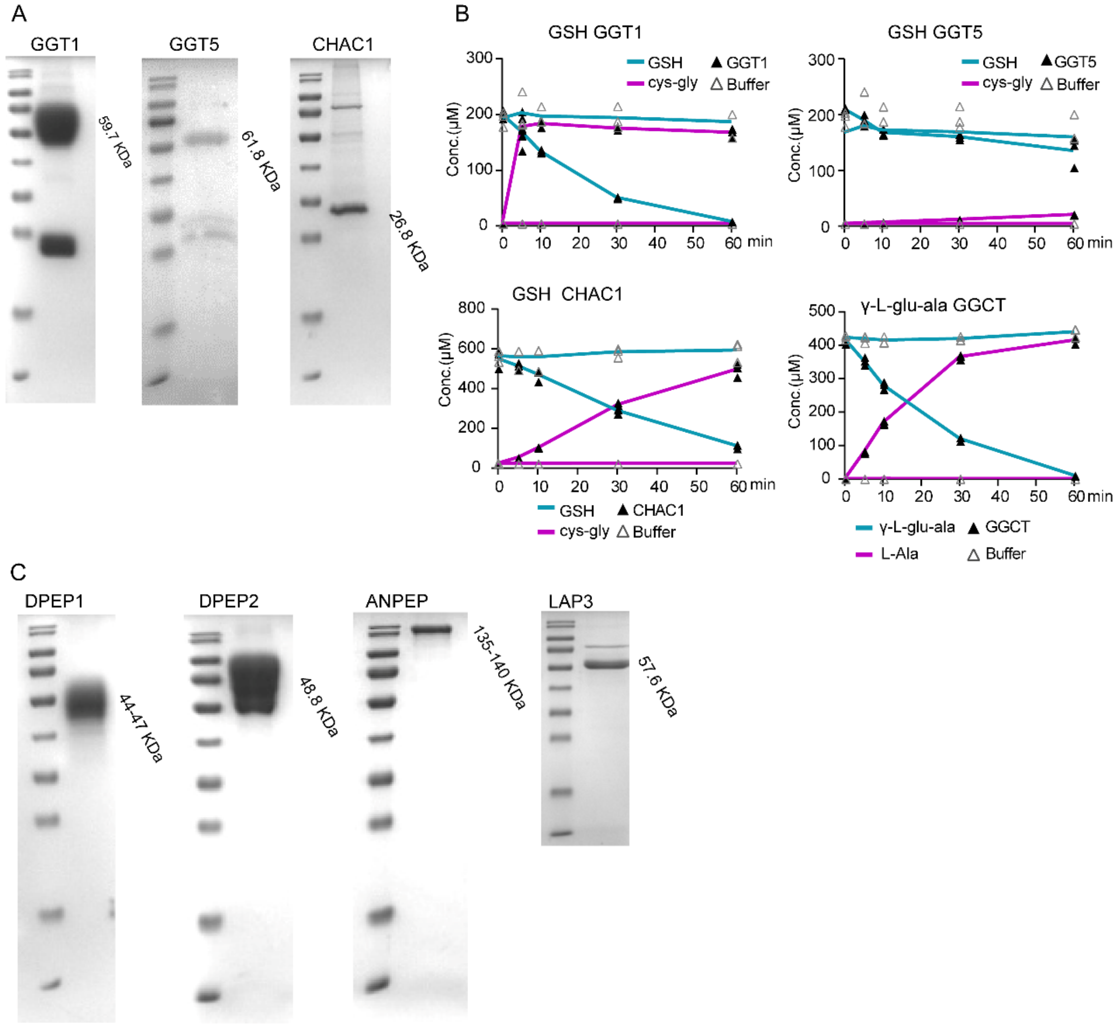

Supplemental Figure 6. The construction of the various human recombinant enzymes and the in vitro assessment of enzymatic activities.

(A) The expression and relative purity of the transpeptidases GGT1, GGT5 and CHAC1 (see methods) was validated by Coomassie blue staining.

(B) Analysis of the in vitro enzymatic conversion of natural substrates to the corresponding indicated product by the recombinant human enzymes, GGT1, GGT5, CHAC1 and GGCT.

(C) The expression and relative purity of the renal dipeptidases DPEP1, DPEP2, LAP3 and ANPEP (see methods) was validated by Coomassie blue staining.

All assays were preformed independently three times. Substrates and proteins were co-incubated at 37ºC and sampling was performed at indicated time points (0, 5, 10, 30, 60 min). In each assay, the buffer was used as non-enzymatic control. All data are presented as Mean ± SEM.

Supplemental Figure 7

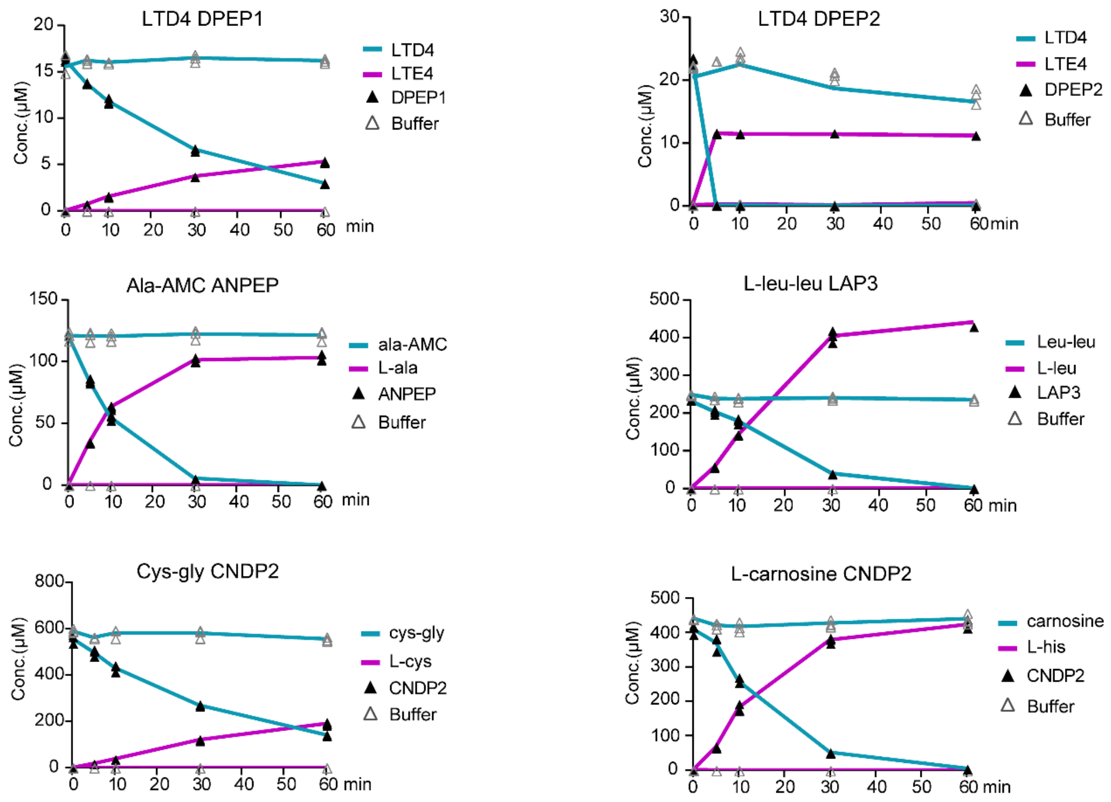

Supplemental Figure 7. The in vitro determinations of enzymatic activities.

Analysis of the in vitro enzymatic conversion of known substrates to the corresponding indicated product by the recombinant human enzymes, DPEP1, DPEP2, CNDP2, LAP3 and ANPEP. All assays were preformed independently three times. Substrates and proteins were co-incubated at 37ºC and sampling was performed at indicated time points (0, 5, 10, 30, 60 min). In each assay, the buffer was used as non-enzymatic control. All data are presented as Mean ± SEM.

Supplemental Figure 8

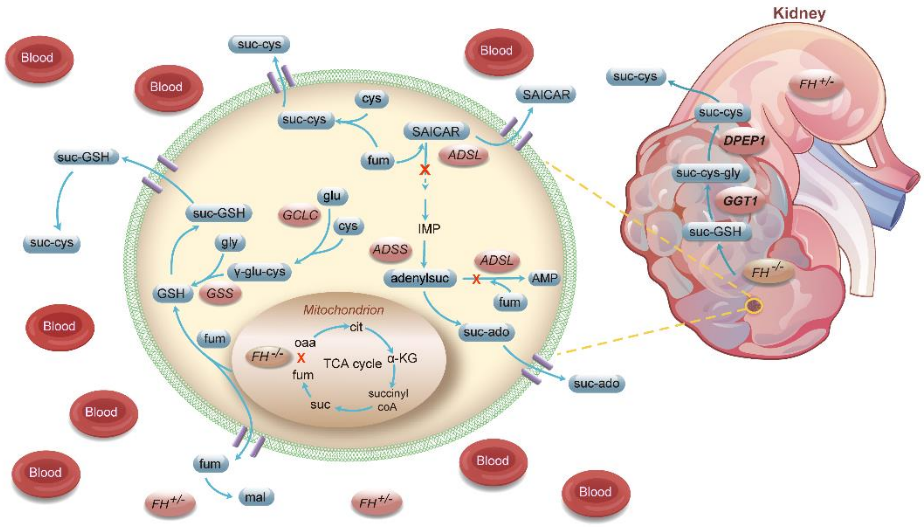

Supplemental Figure 8. A graphic illustration of the compartmentalized production of the identified circulating metabolic biomarkers in FH-deficient RCC.

Supplemental Figure 9

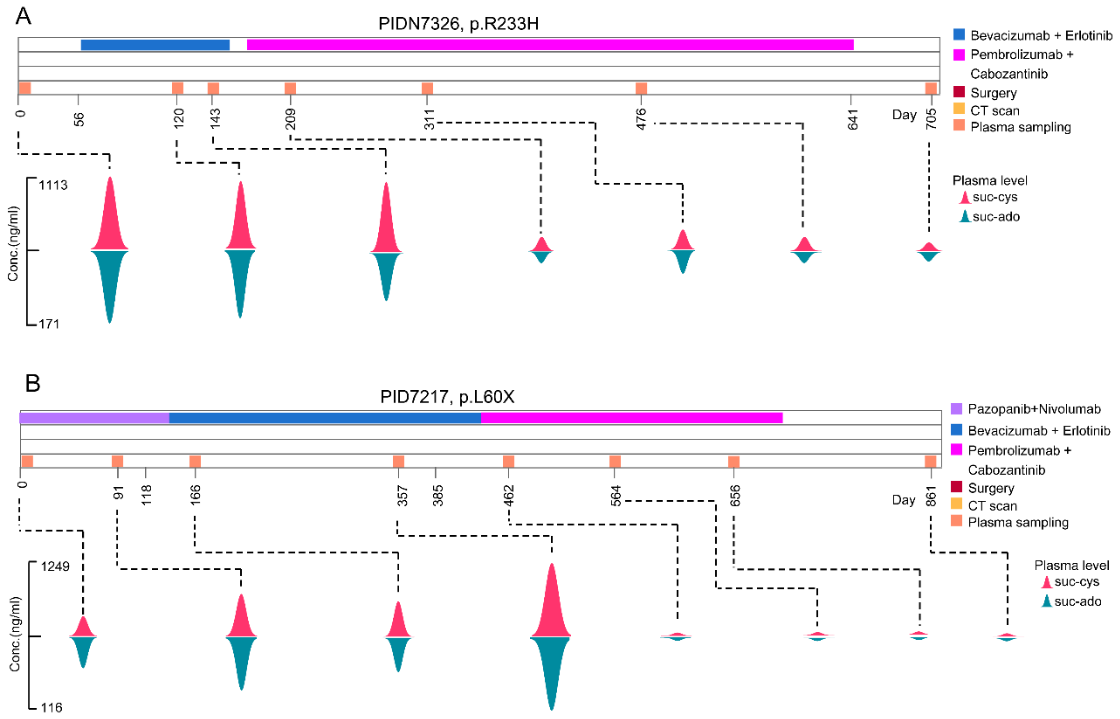

Supplemental Figure 9. Longitudinal monitoring of the dynamics of plasma suc-ado and suc-cys during multiple clinical events in two patients.

PIDN7326 **(A)** and PID7217 **(B).**

Supplemental Table 1. Detailed clinical and pathological characteristics of RCC patients and normal control individuals in the studied cohort SHJTUCMRJ.
Supplemental Table 2. Untargeted metabolomics analyses of human plasma (n=30) in the discovery set.
Supplemental Table 3. Quantification of potential plasma markers (n=238) for ROCAUC analyses in the validation set.
Supplemental Table 4. Details of Recombinant Proteins, Oligonucleotides and Key materials. Related to Methods.
